# Comprehension under threat: outgroup threat predicts perceived accent strength, ease of comprehension, and speaker evaluations

**DOI:** 10.3389/fpsyg.2026.1835739

**Published:** 2026-10-16

**Authors:** Priscilla Lok-chee Shum, Chi-Shing Tse, Stephen C. Wright

**Affiliations:** 1 Department of Management and Marketing, The Hong Kong Polytechnic University, Hong Kong, Hong Kong SAR, China; 2 Department of Educational Psychology, The Chinese University of Hong Kong, Hong Kong, Hong Kong SAR, China; 3 Department of Psychology, Simon Fraser University, Burnaby, BC, Canada

**Keywords:** intergroup threat, language attitudes, nonnative accents, processing fluency, speech perception

## Abstract

Nonnative speakers, influenced by their first language, often produce accented speech that systematically deviates from that of native speakers. Nonnative-accented speech is often judged more harshly than native-accented speech, in part due to increased comprehension difficulty. However, non-linguistic factors like social attitudes can bias perceptions of nonnative accent strength and comprehensibility, which in turn impacts social evaluations of speakers. Grounded in Intergroup Threat Theory, this study examines the relationship between perceived outgroup threat, speech perception, and speaker evaluations within the Hong Kong context. Specifically, in a sample of native Cantonese-speaking Hong Kong undergraduates (*N* = 691), we investigate how perceived threat from Mainland Chinese people influence perceived nonnative accent strength, comprehensibility, and subsequently, social evaluations of native Putonghua speakers, Cantonese-accented Putonghua speakers, native Cantonese speakers, and Putonghua-accented Cantonese speakers. Speakers were rated in terms of competence and warmth, perceived nonnative accent strength, and comprehensibility. Mediation analyses tested the mechanisms through which threat influences speaker evaluation. Perceived outgroup threat was associated with lower competence ratings for Putonghua-accented Cantonese speakers. Threat selectively biased speech perception for Putonghua-accented Cantonese speakers, predicting stronger perceived nonnative accent strength and lower comprehensibility, which mediated the relationship between outgroup threat and speaker evaluations. Robustness checks testing the effects of outgroup threat separately by datasets and accent levels indicated perceived comprehensibility played a more consistent and pivotal role than accent strength in this model. Results suggest listeners engage in social creativity strategies to yield positive ingroup distinctiveness, utilizing linguistic differentiation as a strategic mechanism to penalize speakers from threatening outgroups.

## Introduction

Language attitudes are evaluative responses toward language varieties (Garrett, 2010) that often reflect underlying beliefs about *speakers* of these language varieties (Lambert et al., 1960). Language varieties can be categorized as “standard” or “nonstandard” depending on whether they adhere to or diverge from codified norms that dictate the “correct” use of language (Milroy and Milroy, 1999). Nonnative-accented speech, which refers to “nonpathological speech produced by second-language (L2) learners that differs in partially systematic ways from the speech characteristic of native speakers of a given dialect” (Munro, 1998, p.139), is, by definition, “nonstandard.” On the other hand, while some native accents can be considered nonstandard (e.g., the Cockney accent or the Southern American accent), the standard accent is typically that which is spoken by the majority of native speakers and/or is associated with high social prestige in a given region (e.g., Received Pronunciation in the UK; General American in the US; Giles and Billings, 2004). Extant research shows that nonnative-accented speakers are typically evaluated less favorably than standard native-accented speakers on status (e.g., competence, intelligence) and solidarity (e.g., warmth, sociability) traits (Fuertes et al., 2012; Lindemann and Campbell, 2017), though both native (e.g., Rovetti et al., 2023; Bijvoet, 2026) and nonnative speakers with the same linguistic background (e.g., Hendriks et al., 2016; McKenzie, 2008) sometimes rate nonnative-accented speakers more favorably than those with standard accents. Proposed mechanisms for such biases include stereotyping of nonnative speakers (e.g., Ryan, 1983) and processing difficulty of nonnative speech (e.g., Dragojevic et al., 2017, 2018). The current paper builds on existing work and examines how non-linguistic factors, such as perceived outgroup threat, impact evaluations of nonnative speaker traits both directly and indirectly, through biased perceptions of nonnative accent strength and comprehensibility.

### Mechanisms driving biases against nonnative accents

Biased evaluations against nonnative accents have been attributed to *linguistic stereotyping*: listeners categorize speakers into social groups based on linguistic cues, then ascribe group-linked stereotypes to the speakers (e.g., Ryan, 1983). Given that nonnative-accented speech is often associated with social groups that are lower-status and/or threatening (e.g., immigrants; Birney et al., 2020), native listeners may denigrate nonnative speakers based on stereotypes of these groups. The intergroup perspective, however, fails to explain why evaluations of nonnative-accented speakers become more negative as accent strength increases (e.g., Ryan et al., 1977; but see Ip and Papafragou, 2023 for a specific context where *less proficient* nonnative speakers are favored). To address this, researchers have proposed a complementary, joint mechanism: the disruption of processing fluency (Dragojevic and Goatley-Soan, 2022; Dragojevic et al., 2025).

While processing fluency broadly refers to the ease with which stimuli are processed (e.g., Winkielman et al., 2003), in the current context, it mostly concerns how easily speakers can be understood. According to the *fluency principle*, nonnative-accented speech is harder to understand, and such difficulty elicits negative affect and lower trait evaluations. Indeed, the authors found that processing fluency mediated the relationship between nonnative accent strength and status evaluations of nonnative speakers (Dragojevic et al., 2017, 2018). Past research shows that comprehension aids like subtitles or recording transcripts improved (Dragojevic, 2020) while comprehension barriers such as background noise harmed (Dragojevic and Giles, 2016) status and solidarity evaluations of nonnative speakers.

Processing fluency, as operationalized by the authors (Dragojevic et al., 2017, 2018), is a subjective experience similar to the concept of *comprehensibility* in second language (L2) literature, defined as “perceptions of difficulty in understanding” (Munro and Derwing, 1995, p. 292). L2 research distinguishes between objective (i.e., *intelligibility*) and subjective (i.e., *comprehensibility*) understanding, with the latter more strongly predicted by perceptions of nonnative accent strength (Chau and Huensch, 2025). Critically, subjective judgements of comprehensibility and accent strength are often influenced by non-linguistic factors.

### Intergroup attitudes and biases in speech perception

*Reverse linguistic stereotyping* (Kang and Rubin, 2009) posits that listeners’ beliefs about speakers’ group memberships distort speech perception. Rubin (1992) demonstrated that the same Standard American English passage was judged as more accented and less comprehensible when paired with a picture of an Asian (vs. Caucasian) face (see also Kutlu, 2023; Yi et al., 2013). Likewise, Brown (1992; see also Dragojevic, 2020) showed that perceived country of origin biased participants’ judgements of a nonnative speaker’s language competence. Sheppard et al. (2017) reported that instructors with more negative attitudes towards the language skills of international students rated nonnative speech as less comprehensible, despite transcribing nonnative speech with comparable accuracy. Reid et al. (2019) experimentally manipulated social attitudes by exposing participants to positive or negative comments about nonnative English speakers. However, while older participants showed the expected pattern, with positively-oriented listeners rating nonnative speakers more favorably on accent strength and comprehensibility and negatively-oriented participants, more unfavorably, younger participants rated nonnative speakers favorably regardless of orientation.

In summary, the literature shows that non-linguistic factors impact subjective speech perception, but few studies have directly examined intergroup conflict and speech perception. Building on this work, the current study examines a well-documented *antecedent* of negative social attitudes: perceived outgroup threat (e.g., Stephan et al., 2016).

### Outgroup threat and speaker evaluation

According to the Intergroup Threat Theory (ITT; Stephan et al., 2016), people experience threat from outgroups that they believe can cause harm to their ingroup, which leads to hostility towards these outgroups. The authors outline two fundamental threat types: *realistic threat*, which refers to perceived encroachment on the ingroup’s status, power, and resources; and *symbolic threat,* which refer to perceived challenges to the ingroup’s values, norms, and worldviews (Stephan and Stephan, 2000). The core claim of the ITT, that perceived outgroup threat predicts negative outgroup attitudes, has received much empirical support (Riek et al., 2006). Since language features, especially accents (Kinzler et al., 2009), are important markers of social identity (Giles and Johnson, 1987; Kinzler, 2021), perceived threat should similarly color evaluations of outgroup language varieties. Specifically, we propose that outgroup languages and accents will likely elicit greater negativity from listeners who perceive greater outgroup threat.

Aside from direct effects, perceived outgroup threat may also indirectly impact speaker evaluation via distorted perceptions of nonnative, outgroup speech. When people believe the status of their ingroup is under threat from an outgroup, they may employ strategies to redefine intergroup comparisons in a way that favors the ingroup (i.e., social creativity; Tajfel and Turner, 1979). One type of social creativity strategy is to seek alternate dimensions on which the ingroup compares favorably (e.g., we are less competent, but more likeable; Jackson et al., 1996). Prior research suggests that people emphasize ingroup language markers when interacting with outgroup members to achieve favorable *psycholinguistic distinctiveness* (Giles and Johnson, 1987), especially in the face of outgroup threat. For example, speakers may broaden their ingroup accent (Bourhis and Giles, 1977), switch to their ingroup language (Bourhis et al., 1979), and adhere more strongly to ingroup language norms (Vaes and Wicklund, 2002) when feeling threatened. Moving beyond speech and language production, we propose that the effects of perceived threat extend to speech *perception*. Specifically, listeners may exaggerate perceived *outgroup* speech markers (e.g., accent strength and lack of comprehensibility) in response to threat. Importantly, given the need for positive ingroup distinctiveness, we expect to observe biases in speech perception only in instances where the outgroup is speaking the ingroup language since ingroup speakers are more likely to compare favorably to the outgroup.

### The current study

Hong Kong is a multilingual city, with “biliteracy and trilingualism” policies officially recognizing Cantonese, Putonghua, and English as the three main spoken languages in the region (Bacon-Shone et al., 2025). Cantonese is the primary community language in Hong Kong, with 88.8% reporting Cantonese as their mother tongue, and over 90% using Cantonese to communicate with their spouse, children, parents, and/or friends (Census and Statistics Department, 2019). Putonghua, the official language in China (Chen, 1999), has also increasingly been used as a medium of instruction of the Chinese subject in schools (Bolton and Lee, 2020), with 67.3% of the overall population and 78.7% of students reporting average or above competence in Putonghua (Census and Statistics Department, 2019). While usage of Putonghua in communication with close others remains low (9.5–18.8%), almost a third of employed persons use Putonghua when communicating with external parties and clients (Census and Statistics Department, 2019). Cantonese and Putonghua, though both varieties of Chinese, are not mutually intelligible (Bauer and Benedict, 1997). As such, native Cantonese and Putonghua speakers often speak the other language variety with nonnative accents corresponding to their respective native language (e.g., Ho, 1999; Gu, 2011).

The recent linguistic landscape in Hong Kong has been defined by the tension between Cantonese and Putonghua, which often mirrors contentious intergroup relations between Hong Kong- and Mainland- Chinese (Guo, 2025). Despite its widespread usage, the status of Cantonese is politically contested, with some viewing it as a dialect and others as a vital pillar of the “Hongkonger” identity (Groves, 2010). On the other hand, the promotion of Putonghua is also often resisted and perceived as a strategy for Beijing to exert control over local affairs (Shao, 2016). Large-scale social movements, such as the 2014 Umbrella Movement and the 2019 Anti-Extradition Bill Movement, further polarized language attitudes among Hong Kong locals, resulting in a greater preference for ingroup varieties (Cantonese and the local Hong Kong/Cantonese accent) and heightened rejection of outgroup varieties (Putonghua and Putonghua-accented Cantonese; e.g., Hansen Edwards, 2016, 2021; Shum et al., 2023). These shifts appear intertwined with perceived threat and the othering of Mainland Chinese (Lowe and Tsang, 2017, 2018), though this possibility has yet to be empirically tested.

The current study applies the Intergroup Threat Theory (Stephan et al., 2016) in the domain of intergroup communication and tests whether negativity due to outgroup threat extends to language varieties associated with said outgroup. Further, we explore social creativity as an underlying mechanism that may manifest as biased perception of nonnative outgroup speech. Utilizing data collected from native Cantonese speakers in Hong Kong between 2018 and 2020, we examined the relationship between perceived threat from Mainland Chinese, a salient outgroup in the social movements at the time, and trait evaluations of native- and nonnative-accented speakers of the ingroup (Cantonese) and outgroup (Putonghua) language varieties. We propose that greater perceived threat from the Mainland Chinese would predict more negative attitudes towards language varieties associated with this specific outgroup, resulting in worse competence and warmth evaluations of native speakers of Putonghua and Putongua-accented speakers of Cantonese.

*H1A*: Outgroup Threat will predict lower evaluations of Putonghua-accented Cantonese speakers (but not native Cantonese speakers) by native Cantonese speakers in Hong Kong.

*H1B*: Outgroup Threat will predict lower evaluations of native Putonghua speakers (but not Cantonese-accented Putonghua speakers) by native Cantonese speakers in Hong Kong.

Furthermore, we propose that native Cantonese speakers who perceive greater outgroup threat will exaggerate perceived outgroup speech markers, resulting in higher perceived accent strength and lower comprehensibility ratings of Putonghua-accented Cantonese speakers. Importantly, we do not expect to see the same pattern for ratings of Cantonese-accented speakers of Putonghua, since doing so will yield negative social comparisons for the ingroup speakers against the native Putonghua-speaking outgroup.

*H2*: Outgroup threat will predict higher perceived accent strength ratings of Putonghua-accented Cantonese speakers by native Cantonese speakers in Hong Kong.

*H3*: Outgroup threat will predict lower comprehensibility ratings of Putonghua-accented Cantonese speakers by native Cantonese speakers in Hong Kong.

Finally, we propose that biased speech perception serves as the mechanism that propels (and perhaps justifies) negative social evaluations of Putonghua-accented Cantonese speakers. Thus we expect perceptions of accent strength and comprehensibility to serially mediate the effect of outgroup threat on trait evaluations of Putonghua-accented Cantonese speakers.

*H4*: The relationship between outgroup threat and attitudes towards Putonghua-accented Cantonese speakers is serially mediated by greater perceived accent strength and lower ease of comprehension.

## Method

Data from three datasets collected by the first author in the years 2018–2020^1^ were analyzed. A portion of the 2018 and 2019 datasets was previously reported in a paper that investigated the impact of political movements on language attitudes (Shum et al., 2023)^2^ while the 2020 dataset has not been reported in any prior publications. Although the current study also examines speaker evaluations, the predictor (outgroup threat) and mediating variables (perceived accent strength and comprehensibility) are wholly novel and share no overlap with the prior paper.

### Participants

A total of 691 Hong Kong native Cantonese-speaking undergraduates (228 men, 463 women; Mean age = 19.87, *SD* = 1.75) provided complete responses. In return for completing the study, participants were entered into a lucky draw of HK$300 (approximately US$39). Most participants reported moderate (3) or above moderate proficiency in Putonghua (88.20%; *M* = 3.35/5, *SD* = 0.83) and rated their own accent strength as moderate (3) or below when speaking Putonghua (82.3%; *M* = 2.64/5, *SD* = 0.77). Most participants reported that they used Putonghua “sometimes” (3) or less frequently in their daily lives (93.3%; *M* = 2.24/5, *SD* = 0.78). Over 98% of participants identified moderately (3) or more strongly as *“Hongkongers”* (*M* = 4.68/5, *SD* = 0.66).

### Procedure

All data were collected online using Qualtrics. Participants were randomly assigned to listen to 8 speakers (4 men; 4 women) who either spoke Cantonese (*N* = 337) or Putonghua (*N* = 354). These speakers varied in terms of their accent strength (2 native; 2 mild, 2 moderate, and 2 strong nonnative; see Appendix A for pilot results). Nonnative speakers in the Cantonese condition were native Putonghua speakers from Mainland China who spoke Putonghua-accented Cantonese. Nonnative speakers in the Putonghua condition were native Cantonese speakers from Hong Kong who spoke Cantonese-accented Putonghua. All speakers read out the same short passage about a biannual city-wide sports event^3^ (see Appendix A). After listening to each speaker, participants provided ratings of competence, warmth and accent strength on 5-point scales, and ease of comprehension on 7-point Likert scales. At the end, participants responded to 15 items on 5-point Likert scales that measured perceived outgroup threat from Mainland Chinese,^4^ and completed demographic measures, including gender, age, social identity, and language proficiencies and use (see Appendix E for full measures).

### Speaker ratings

#### Speaker evaluation

After listening to each audio track, participants rated the speaker’s competence and warmth (Cuddy et al., 2009). Participants rated the speaker on four Competence adjectives (*competent, confident, skillful, capable*) and four Warmth adjectives (*friendly, good-natured, warm, sincere*), using 5-point Likert scales anchored by 1 = “*extremely disagree*” and 5 = “*extremely agree*.” Both scales had good reliability (Competence: α = 0.93; Warmth: α = 0.91).

#### Accent strength

Participants rated the accent of each speaker (“The speaker has…”; “The speaker’s accent is…”) on 5-point Likert scales (1 = “no accent at all” and 5 = “a very strong accent”; 1 = “*very standard*” and 5 = “*very non-standard*”). The two items had good reliability (α = 0.84).

#### Comprehensibility

Participants rated the comprehensibility of each speaker with five items (e.g., “I don’t have any problems understanding the speaker” and “I have to listen very closely to understand what the speaker says - R”) on 5-point Likert scales (1 = “*extremely disagree*” and 5 = “*extremely agree*”). The five items had good reliability (α = 0.92).

### Participant measures

#### Perceived outgroup threat

Participants filled out a 15-item Intergroup Threat scale (Stephan et al., 1999) modified to measure perceived threat of Mainland Chinese. Example items include “Mainland Chinese immigrants should learn to conform to the rules and norms of Hong Kong society as soon as possible after they arrive” (7 items; Symbolic Threat) and “Mainland Chinese immigrants get more from this city than they contribute” (8 items; Realistic Threat). Participants rated each item on a 5-point Likert scale (1 = “*completely disagree*”; 5 = “*completely agree*”). The 15-item scale had good reliability (α = 0.86).^5^

#### Language proficiency

Participants reported their Cantonese and Putonghua proficiencies in response to the item (“Your Cantonese/Putonghua proficiency is …”) on a 5-point Likert scales (1 = *“extremely low”;* 5 = *“extremely high”*).

#### Self-reported accentedness

Participants reported their own accent strength when speaking Cantonese and Putonghua by responding to two items (“*I have…*”; “My accent is…”) on 5-point Likert scales (1 = “no accent at all” and 5 = “a very strong accent”; 1 = “*very standard*” and 5 = “*very non-standard*”). The two items had acceptable reliability for Putonghua (α_pth_ = 0.78) but poor reliability for Cantonese (α_cant_ = 0.57). However, to maintain comparability, the two items were averaged to form an Accentedness score for each language variety.

#### Social identification

Participants responded to the item (“You describe yourself as a… [Hongkonger/Chinese person]”) on 5-point Likert scales (1 = *“extremely disagree”;* 5 = *“extremely agree”*).

## Results

Since participants rated multiple speakers, we used multilevel linear regressions with ratings (Level 1; *N* = 5,528) nested within participants (Level 2; *N* = 691) to test Hypotheses 1–3 (see below for the details of a moderated serial mediation model to test Hypothesis 4). For interpretability, nonnative accents (mild, moderate, strong^6^) were collapsed into a single nonnative category, thus yielding a dichotomous Speaker Accent variable. Outgroup Threat was grand-mean-centered prior to inclusion in the model. All analyses were conducted using the lme4 package (Bates et al., 2015) in R 4.4.0 (R Core Team, 2024). All models were estimated using restricted maximum likelihood (REML). *p*-values for fixed effects were estimated via Satterthwaite’s degrees of freedom method with the lmerTest package (Kuznetsova et al., 2017). Standardized regression coefficients were obtained using the *standardize_parameters()* command in the effectsize package (Ben-Shachar et al., 2020). Normality of residuals and homoskedasticity was confirmed via visual inspection of QQ-plots and histograms of the residuals, and residual-vs-fitted plots, respectively. Simple slopes were estimated and compared using the *emtrends()* command in the emmeans package (Lenth and Piaskowski, 2026). Reported *p*-values and confidence intervals were adjusted for multiple comparisons with the Sidak correction.

### Speaker evaluation

For competence and warmth ratings, we included Speaker Accent (Native vs. Nonnative), Language Variety (Cantonese vs. Putonghua), Perceived Outgroup Threat, as well as all interactions as fixed effects.^7^ To examine cross-level interactions (e.g., Language × Accent × Threat), we specified random effects for Speaker Accent (Heisig and Schaeffer, 2019) for participant and crossed random intercepts for participant and speaker,^8^ and controlled for participants’ gender, age, language proficiencies, and social identification, dataset, and speakers’ gender. For brevity, we only report the critical interaction and simple effects. Full regression results are presented in Table 1. Graphical representation of interaction effects is presented in Figure 1.

**Table 1:** Summary of multilevel regression results for model predicting ratings of speaker competence, warmth, accent strength, and ease of comprehension.

Effect	Competence	Warmth	Accent strength	Compre-hensibility
Fixed effects				
(Intercept)	3.20*** (0.47)	2.97*** (0.34)	1.62** (0.58)	4.60*** (0.54)
Accent (Nonnative)	−1.09** (0.41)	−0.43* (0.17)	2.29*** (0.50)	−1.89*** (0.50)
Language (Putonghua)	0.14** (0.04)	−0.28*** (0.05)	0.36*** (0.05)	−0.15*** (0.04)
Outgroup threat	0.12* (0.06)	0.07 (0.06)	−0.05 (0.08)	0.05 (0.06)
Accent (Nonnative) × Language (Putonghua)	0.01 (0.05)	0.34*** (0.05)	−0.93*** (0.06)	0.71*** (0.05)
Language (Putonghua) × Outgroup threat	−0.16*	−0.20*	0.05	−0.09
	(0.08)	(0.09)	(0.10)	(0.07)
Accent (Nonnative) × Outgroup threat	−0.32*** (0.06)	−0.11 + (0.06)	0.23** (0.08)	−0.34*** (0.06)
Accent (Nonnative) × Language (Putonghua) × Outgroup threat	0.32*** (0.09)	0.31*** (0.09)	−0.24* (0.11)	0.38*** (0.09)
Demographic controls				
Age	−0.00 (0.01)	−0.01 (0.01)	0.00 (0.01)	0.01+ (0.01)
Gender (Woman)	−0.03 (0.03)	0.02 (0.03)	0.02 (0.04)	−0.05+ (0.03)
Cantonese proficiency	−0.01 (0.03)	0.04 (0.03)	−0.03 (0.03)	0.02 (0.03)
Putonghua proficiency	0.08** (0.02)	0.06* (0.03)	−0.01 (0.03)	0.06* (0.02)
Cantonese accentedness	0.08** (0.03)	0.06+ (0.03)	0.00 (0.03)	−0.00 (0.03)
Putonghua accentedness	0.04 (0.03)	0.05+ (0.03)	0.05 (0.03)	−0.02 (0.03)
Social identification				
Hongkonger identity	0.05+ (0.02)	0.02 (0.03)	−0.01 (0.03)	0.07** (0.02)
Chinese identity	0.00 (0.01)	0.01 (0.02)	0.03 (0.02)	0.02 (0.01)
Dataset (2019)	0.06+ (0.04)	0.03 (0.04)	−0.05 (0.04)	0.05 (0.04)
Dataset (2020)	0.08* (0.03)	0.05 (0.04)	−0.08* (0.04)	0.09** (0.03)
Speaker gender (Male)	−0.06 (0.35)	−0.07 (0.15)	0.22 (0.44)	−0.18 (0.43)

Random effects				
	Var (SD)	Var (SD)	Var (SD)	Var (SD)
Intercept | SUBJECT	0.11 (0.33)	0.18 (0.42)	0.30 (0.55)	0.11 (0.32)
Nonnative accent | SUBJECT	0.15 (0.38)	0.17 (0.41)	0.37 (0.61)	0.17 (0.42)
Intercept | SPEAKER	0.25 (0.50)	0.04 (0.21)	0.38 (0.62)	0.37 (0.61)
Residual	0.37 (0.61)	0.36 (0.60)	0.41 (0.64)	0.32 (0.57)
Obs.	5,528	5,528	5,528	5,528

*** < 0.001, ** < 0.01, * < 0.05, ^+^ < 0.10.

**Figure 1:**
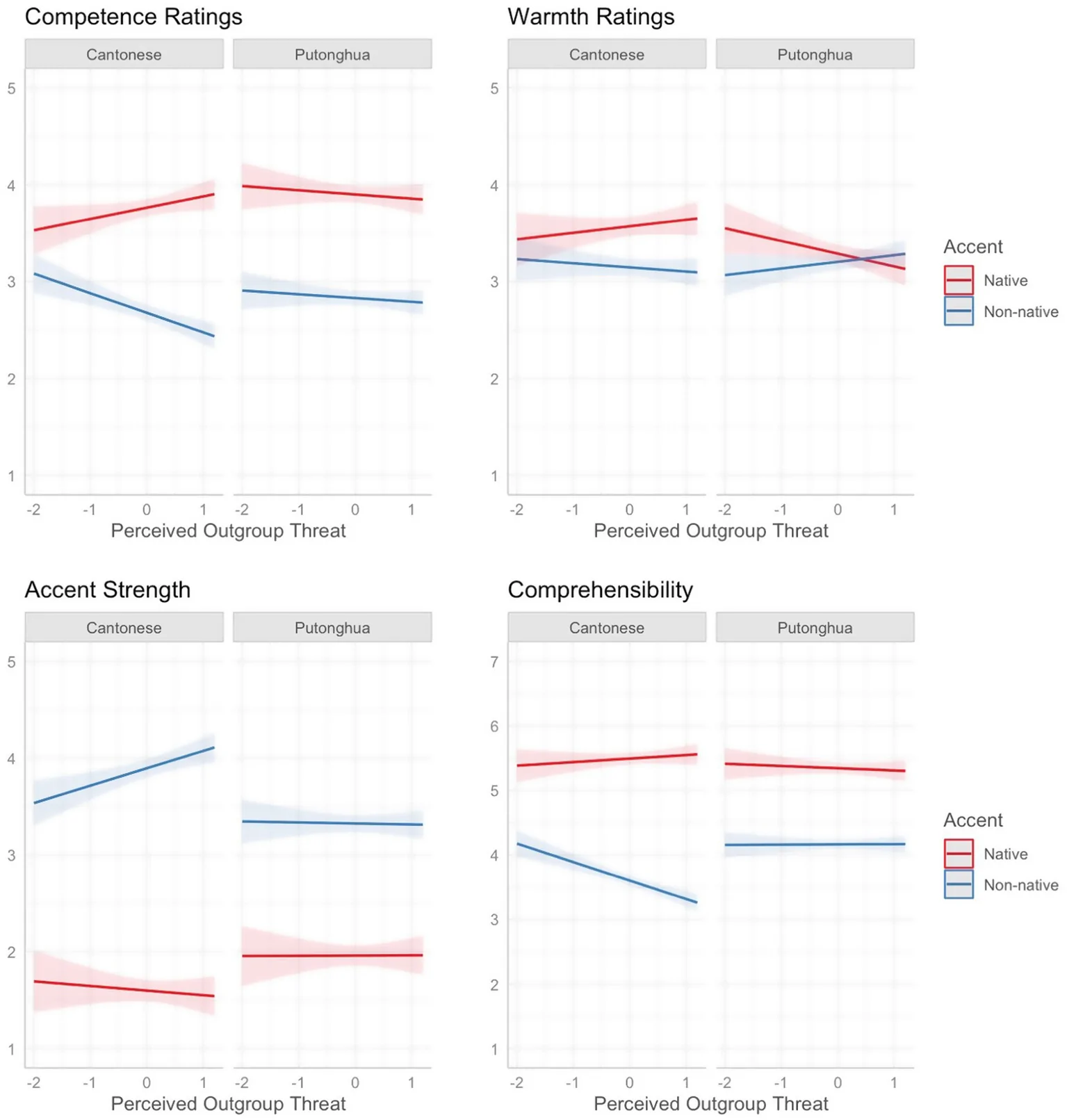
Speaker evaluation and speech perception variables by perceived outgroup threat, language variety, and speaker accent type.

#### Competence

The critical three-way Threat × Speaker Accent × Language interaction was significant, *β* = 0.19, *B* = 0.32, *SE* = 0.09, *95% CI* [0.15, 0.50], *t*(687.00) = 3.63, *p* < 0.001. Simple 2-way interaction contrasts show a significant Accent × Threat interaction for Cantonese, *B* = 0.32, *SE* = 0.06, *95% CI* [0.15, 0.49], *z* = 5.03, *p* < 0.001, but not Putonghua speakers (*p* = 1.00). Analyses of simple slopes show that threat predicted lower competence ratings of Putonghua-accented Cantonese speakers, *B* = −0.20, *SE* = 0.05, *95% CI* [−0.32, −0.09], *z* = −4.39, *p* < 0.001 (H1A supported), but not native Putonghua speakers, *B* = −0.04, *SE* = 0.06, *95% CI* [−0.19, 0.10], *z* = −0.75, *p* = 0.91 (H1B not supported). No other simple slopes reached significance (*p*s > 0.17).

#### Warmth

The three-way Threat × Speaker Accent × Language interaction was significant, *β* = 0.22, *B* = 0.31, *SE* = 0.09, *95% CI* [0.13, 0.49], *t*(687.00) = 3.44, *p* < 0.001. Simple 2-way interaction contrasts show a significant Accent × Threat interaction for Putonghua, *B* = –0.20, *SE* = 0.06, *95% CI* [−0.37, −0.03], *z* = −3.16, *p* = 0.010, but not Cantonese speakers (*p* = 0.42). Analyses of simple slopes show that threat weakly predicted lower warmth ratings of native Putonghua speakers, but the effect was no longer significant after adjusting for multiple comparisons, *B* = −0.13, *SE* = 0.06, *95% CI* [−0.29, 0.003], *z* = −2.08, *p* = 0.14 (H1B not supported). Threat also did not predict warmth of Putonghua-accented Cantonese speakers, *B* = −0.04, *SE* = 0.05, *95% CI* [−0.17, 0.08], *z* = −0.84, *p* = 0.87 (H1A not supported). No other simple slopes reached significance (*ps* > 0.52).

Perceived threat from Mainland Chinese predicted competence ratings of Putonghua-accented Cantonese speakers only. However, supplementary analyses suggest that this effect lacks robustness and is isolated to the 2018 dataset (see Appendix F). Furthermore, while perceived threat predicted a smaller *difference* in warmth ratings between native and Cantonese-accented Putonghua speakers (as evidenced by the significant simple 2-way contrast), threat did not significantly predict ratings of native Putonghua speakers. Therefore, Hypothesis 1 receives limited support.

### Speech perception

The same multilevel linear regression models as above were tested for Accent Strength and Ease of Comprehension. Full results and interactions are presented in Table 1 and Figure 1.

#### Accent strength

The three-way Threat × Speaker Accent × Language interaction was significant, *β* = −0.10, *B* = −0.24, *SE* = 0.11, *95% CI* [−0.46, −0.02], *t*(687.00) = −2.10, *p* = 0.036. Simple 2-way interaction contrasts show a significant Accent × Threat interaction for Cantonese speakers, *B* = −0.23, *SE* = 0.08, *95% CI* [−0.44, −0.01], *z* = −2.80, *p* = 0.03, but not Putonghua speakers (*p* = 1.00). Simple slopes show that threat predicted higher accent strength ratings of Putonghua-accented Cantonese speakers, *B* = 0.18, *SE* = 0.05, *95% CI* [0.05, 0.31], *z* = 3.36, *p* = 0.003 (H2 supported), but not native Cantonese speakers (*p* = 0.95). No other simple slopes reached significance (*ps* > 0.95).

#### Comprehensibility

The three-way Threat × Speaker Accent × Language interaction was significant, *β* = 0.19, *B* = 0.38, *SE* = 0.09, *95% CI* [0.21, 0.55], *t*(687.00) = 4.29, *p* < 0.001. Simple 2-way interaction contrasts show a significant Accent × Threat interaction for Cantonese speakers, *B* = 0.34, *SE* = 0.06, *95% CI* [0.18, 0.51], *z* = 5.41, *p* < 0.001, but not Putonghua speakers (*p* = 0.99). Simple slopes show that threat predicted lower comprehensibility ratings of Putonghua-accented Cantonese speakers, *B* = −0.29, *SE* = 0.05, *95% CI* [−0.40, −0.17], *z* = −6.17, *p* < 0.001 (H3 supported), but not native Cantonese speakers (*p* = 0.80). No other simple slopes reached significance (*ps* > 0.96).

In summary, Hypothesis 3 was supported. Hong Kong participants who perceived greater threat from Mainland Chinese rated Putonghua-accented Cantonese speakers significantly lower on comprehensibility. While perceived threat also predicted significantly higher accent strength ratings, robustness checks show that the effect is restricted to the 2018 dataset (Appendix F) and mild nonnative-accented Putonghua speakers only (Appendix H). Therefore, the findings in support of Hypothesis 2 must be interpreted with caution.

### Moderated serial mediation

To test for moderated serial mediation (Hypothesis 4), we took the unweighted average of within-participant ratings of all six nonnative speakers within each language variety to obtain single scores for accent strength, ease of comprehension, competence, and warmth per language. Doing so removed dependencies between data points, allowing us to test moderated mediation models using the PROCESS macro (model 85; Hayes, 2022) in R (version 4.4.0).^9^ We tested moderated mediation models with Perceived Outgroup Threat as the predictor, Language Variety (Cantonese vs. Putonghua) as the moderator,^10^ Accent Strength and Ease of Comprehension as the mediators, and Competence and Warmth as the outcome variables. We included participant age, gender, and dataset (dummy-coded) as controls. Estimated indices of moderated mediation and conditional indirect effects are presented in Table 2. Full regression results are presented in Appendix C.

**Table 2:** Index of moderated mediation and conditional indirect effects by language variety.

Mediator(s)	Outcome	Index of mediated moderation (SE)	95% CI	Conditional indirect effects			
				Cantonese	Putonghua		
				*B* (SE)	95% CI	*B* (SE)	95% CI
Accent → Compre-hensibility	Competence	0.**03 (0.02)**	**[0.01, 0.07]**	**−0.04 (0.01)**	**[−0.07, −0.01]**	0.002 (0.01)	[−0.02, 0.02]
	Warmth	0.**03 (0.01)**	**[0.004, 0.06]**	**−0.03 (0.01)**	**[−0.05, −0.01]**	0.002 (0.001)	[−0.02, 0.02]
Accent	Competence	0.**02 (0.01)**	**[0.002, 0.05]**	**−0.02 (0.01)**	**[−0.04, −0.004]**	0.001 (0.01)	[−0.02, 0.01]
	Warmth	0.01 (0.01)	[−0.01, 0.03]	−0.01 (0.01)	[−0.03, 0.01]	−0.001 (0.004)	[−0.01, 0.01]
Compre-hensibility	Competence	0.**07 (0.02)**	**[0.03, 0.12]**	**−0.08 (0.02)**	**[−0.12, −0.05]**	−0.01 (0.01)	[−0.04, 0.02]
	Warmth	0.**06 (0.02)**	**[0.03, 0.10]**	**−0.06 (0.02)**	**[−0.10, −0.03]**	−0.01 (0.01)	[−0.03, 0.02]

Significant effects in bold.

The index of moderated mediation for the hypothesized path (Threat → Accent → Comprehension → Outcome) was significant for both Competence, *B* = 0.03, *SE* = 0.02, *95% CI* [0.01, 0.07], and Warmth, *B* = 0.03, *SE* = 0.01, *95% CI* [0.004, 0.06], since 95% confidence intervals of the indices did not include 0 (H4 supported). For nonnative Cantonese speakers, conditional indirect effects of threat via Accent and Comprehension were significant for both Competence, *B* = −0.04, *SE* = 0.01, *95% CI* [−0.07, −0.01], and Warmth, *B* = −0.03, *SE* = 0.01, *95% CI* [−0.05, −0.01]. For nonnative Putonghua speakers, indirect paths via Accent and Comprehension were non-significant for both variables. Figure 2 presents effects and significance levels of each path in the mediation models for nonnative Cantonese and Putonghua speakers, respectively.

**Figure 2:**
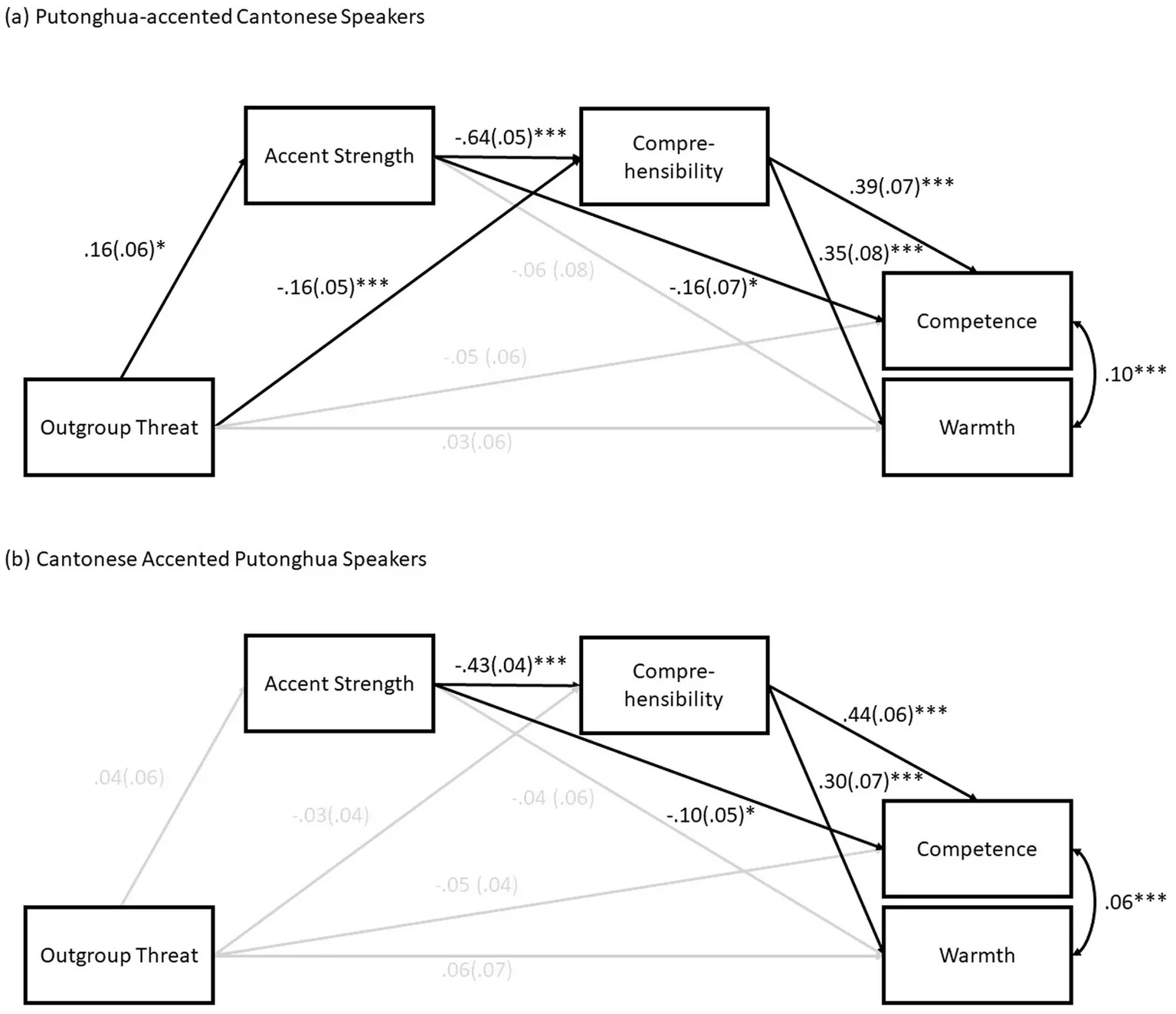
Path diagrams illustrating the serial mediation model for nonnative Cantonese and Putonghua speakers respectively. Serial mediation models were fit using lavaan (Rosseel, 2012) in R 4.4.0 (R Core Team, 2024) for Putonghua-accented Cantonese and Cantonese-accented Putonghua speakers. We tested three models in the multigroup analyses: a fully free model where all parameters were permitted to vary across language varieties, a partially restricted model where only threat pathways were allowed to vary, and a fully restricted model where all pathways were set to be the same. We compared the nested models against each other using likelihood ratio tests and found that the fully free model had significantly better fit than the partially free model, χ^2^(5) = 18.04, *p* = 0.003, which in turn had significantly better fit than the fully restricted model, χ^2^(4) = 9.57, *p* = 0.05. The figure presents estimates of the fully free model. Bootstrapping with 5,000 samples was used to estimate standard errors (presented in brackets). *** < 0.001, ** < 0.01, * < 0.05, non-significant paths in gray.

The indirect effects of perceived outgroup threat on speaker evaluation were mediated through accent strength and ease of comprehension for nonnative Cantonese speakers, but not for nonnative Putonghua speakers. However, the results show that the indirect effect via comprehensibility alone is substantially stronger, suggesting that comprehensibility may account for the bulk of the serial indirect effect. Coupled with the lack of robustness of the effects of perceived threat on accent strength across datasets and accent level, evidence in support of the accent strength-comprehensibility pathway (Hypothesis 4) is limited.

## Discussion

The purpose of the current study was to examine the relationship between perceived outgroup threat, speech perception, and speaker evaluations for ingroup and outgroup language varieties amongst native Cantonese speakers in Hong Kong. Three main findings emerged.

First, perceived outgroup threat was associated with more negative evaluations of outgroup-related speech, but the effect was dimension-specific. Perceived threat predicted lower competence ratings of Putonghua-accented Cantonese speakers, but only in the 2018 dataset. In times of heightened threat, such as during political turmoil in 2019 and 2020, this effect was surprisingly absent. One potential reason is the overall heightened intergroup tensions in 2019 and 2020, which likely resulted in denigration of the Putonghua-accented Cantonese speaker regardless of perceived threat. The denigration of outgroup members in the face of outgroup threat is consistent with the Intergroup Threat Theory (Stephan et al., 2016), indicating that negative sentiment towards threatening outgroups extended to speakers with outgroup linguistic markers. Surprisingly, however, threat was not associated with warmth ratings of Putonghua-accented Cantonese speakers, despite warmth typically being more strongly associated with intergroup competition (Fiske et al., 2002).

The null finding for warmth could be attributed to a counteracting effect of perceived assimilation (on the part of the speakers). Language choice is often a source of friction in Hong Kong: Mainland Chinese are seen as reluctant to speak Cantonese, which is attributed to a lack of willingness to integrate into the local culture (e.g., Leung and Yu, 2026). Prior research shows that perceived assimilation was associated with more positive perceptions of immigrants (e.g., Alcott and Watt, 2017; Koc and Anderson, 2018), and that people who perceived greater threat tended to support assimilation and oppose multiculturalism (e.g., Callens et al., 2018). Speaking Cantonese may be perceived as an attempt to assimilate with Hong Kong culture, which, given its focus on challenges to ingroup rules and norms, would be received especially well by Hong Kong participants who perceive greater symbolic threat from the Mainland Chinese. Coupled with the fact that Cantonese-accented Putonghua speakers were rated as substantially less competent (hence also assuaging realistic threat concerns; Rios et al., 2018), threatened participants may have employed a kind of “reverse social creativity”, in which higher-status groups attribute *more* warmth to outgroups as a way to appear non-discriminatory and maintain the status quo (van Bezouw et al., 2021; Yzerbyt and Cambon, 2017).

Perceived threat did not predict competence nor warmth ratings of native Putonghua speakers, but was associated with a smaller *difference* in warmth ratings of native vs. Cantonese-accented Putonghua speakers. On the other hand, native Putonghua speakers were rated as more competent than Cantonese-accented Putonghua speakers regardless of perceived threat. This pattern is consistent with one of the social creativity strategies proposed by Tajfel and Turner (1979), in which group members change the comparison dimension from one on which the ingroup compares unfavorably to one that better favors the ingroup (van Bezouw et al., 2021). Given that native Putonghua speakers were undeniably more competent in a context where the salient cross-group comparison is based on Putonghua proficiency, participants who perceived greater outgroup threat may have instead focused on ingroup warmth as a way to minimize the unfavorable comparison between the ingroup and outgroup speakers.

Second, perceived threat selectively predicted stronger perceived accent strength and lower comprehensibility ratings of Putonghua-accented Cantonese speakers, but not of Cantonese-accented Putonghua speakers. In other words, native Cantonese speakers only accentuated differences on Cantonese proficiency, which is central to ingroup identity and produced favorable ingroup comparisons. However, while the effects were strong and robust for comprehensibility, the effects on accent strength are specific to dataset and mild accents, suggesting that the former plays a more pivotal role. Apart from being consistent with social creativity strategies, this pattern also aligns with evidence that threat motivates increased intergroup distancing specifically on valued group attributes (Grant, 1992, 1993; Lalonde, 2002). Native Putonghua speakers were not penalized on accent strength or comprehensibility, suggesting that the observed pattern cannot be explained by a general increase in negativity towards outgroup voices.

Third, we found that perceived accent strength and ease of comprehension fully mediated the relationship between outgroup threat and evaluations of Putonghua-accented Cantonese speakers, which suggests (positive) linguistic differentiation could be a potential mechanism by which native Cantonese speakers penalized nonnative Cantonese speakers from the threatening outgroup. The accent-to-comprehension link also substantiates the fluency principle (Dragojevic et al., 2017, 2018; Dragojevic, 2020; Dragojevic and Giles, 2016): listeners’ attitudes towards accented speech are partially (competence) or fully (warmth) determined by how easily accented speech is processed. However, given the fact that the predictors, mediators, and outcomes were measured out of order (with outgroup threat measured last) and in quick succession, the serial causal chain cannot be established. While we tested serial mediation via accent strength and comprehensibility, alternate models with different sequential order and directionality is also plausible. Indeed, given the relative strength of the indirect comprehensibility path compared to the full serial mediation path, there is a possibility that comprehensibility may play a more important role than accent strength perceptions.

### Implications

Our study adds to the literature on the biasing effect of non-linguistic factors on speech perception, underscoring the need to consider listener attitudes in assessments of nonnative speech. Such biases may be especially problematic in learning contexts, where students may be evaluated unfairly due to factors unrelated to actual performance. Prior studies show that comprehension aids improve listeners’ evaluations of nonnative speakers (Dragojevic, 2020). Familiarity with and prior exposure to nonnative accents also improve comprehension (e.g., Gass and Varonis, 1984; Bradlow and Bent, 2008) and speaker evaluation (Rovetti et al., 2023). These findings suggest interventions that improve comprehensibility, whether through aids or increased familiarity of nonnative speech, could mitigate bias.

Contact with nonnative speakers can increase familiarity with their accents, which in turn reduces bias. Indeed, people who live in more diverse locales (Kutlu et al., 2022a) and have more diverse social networks (Kutlu et al., 2022b) were less susceptible to reverse linguistic stereotyping. Critically, positive intergroup contact also predicts lower perceived intergroup threat (e.g., Pettigrew et al., 2007; Aberson, 2015), especially in contexts where equality (Kende et al., 2018) and integration (Green et al., 2020) is encouraged. Taken together, *bilingual* interaction between native Cantonese and Putonghua speakers could potentially improve language attitudes via dual pathways of reduced threat and increased processing fluency. Given the increasing number of cross-boundary students from Mainland China attending secondary schools in Hong Kong (Education Bureau, 2025), bilingual instruction in the classroom (e.g., Wright and Tropp, 2005) may serve as a promising way to simultaneously reduce linguistic bias and improve intergroup relations. Future experimentation should test this proposition.

### Limitations and future directions

The current study has a number of limitations. First, our study design limits the certainty of conclusions that can be drawn. The study is cross-sectional, thus causality cannot be established; we did not measure perceived ethnicity of speakers,^11^ and thus cannot definitively attribute speaker evaluations to intergroup processes; and we only examined one nonnative accent in the current study, thus it remains unclear if the downgrading of nonnative Cantonese speakers reflects group-specific bias towards Putonghua-accented Cantonese or a general tightening of ingroup boundaries that excludes *all* nonnative Cantonese speakers from the ingroup. Future work should experimentally manipulate threat and/or comprehensibility in a mediation-by-moderation design. Perceived social group membership should be included as a manipulation check or tested as a moderator to clearly delineate intergroup and non-intergroup effects. Nonnative variants of Cantonese spoken by other outgroups (e.g., Americans or South Asians) should be included to clarify exact processes driving nonnative speaker evaluation.

Furthermore, the choice of recording content, small number of speakers, analytical strategy, and the unique sociolinguistic context in which the study was conducted also limit generalizability of our results. Since all speakers read out the same passage, we could not test whether different types of content (e.g., formal vs. informal) would moderate the evaluation of native and nonnative accents; each accent level was represented by two (young adult) speakers only, throwing into question whether the same patterns would be observed for speakers with different characteristics; we also collapsed across nonnative accent levels in our main analyses, which may obscure important differences in the evaluation of nonnative speakers with varying levels of accent strength; and the data used in this study was also conducted during a period of major societal upheaval, during which perceived outgroup threat was likely elevated beyond typical levels. As a result of these factors, the findings in the current study may represent a snapshot in time and place that do not extend to other intergroup contexts. Future studies should broaden the scope of investigation to cover less contentious intergroup relations, in a larger variety of social situations, with finer grained analyses of speakers with varying accent strength.

## Conclusion

Our study extends our understanding of intergroup threat in the domain of communication and speech perception. We integrate the intergroup and processing fluency accounts, demonstrating that social attitudes shape not only what listeners think about speakers but also how speech is perceived and processed. Our results suggest that perception of threat predicts strategic denigration of outgroup speakers in ways that yield maximal positive ingroup differentiation. The key role that comprehensibility plays in this process suggests interventions that reduce perceived threat and increase processing fluency hold promise for improving evaluations in linguistically diverse societies.

## Data Availability

The raw data supporting the conclusions of this article will be made available by the authors, without undue reservation.

## Footnotes

1 Perceived outgroup threat varied across datasets: participants in the 2019 and 2020 dataset reported significantly greater perceived threat than those in the 2018 dataset. We conducted additional analyses to test whether Dataset moderated our key effects. Results indicate that the effects of threat on speaker warmth and comprehensibility were invariant across datasets, but the effects of threat on speaker competence and accent strength were substantially stronger in the 2018 dataset (see Appendix F).

2 In previously published work, we focused on replicating differences found in two prior datasets (2013, 2015) and thus only compared and reported ratings of female native- and mild nonnative-accented speakers (matching speech stimuli used in the other datasets). As a result, ratings of all male speakers and the moderate and strong “nonnative” female speakers from the 2018 and 2019 datasets have not been reported in any prior publications.

3 The passage was adapted from a government website about the Hong Kong Games. To avoid priming a Hongkonger identity, all mentions of Hong Kong were removed. The passage was written in Standard written Chinese (SWC), which is based on Putonghua but pronounced in Cantonese in Hong Kong (Li, 2022). SWC is taught in schools as part of the biliteracy and trilingual language policy and is commonly used in more formal contexts (Cheng and Tang, 2016). Given its proximity to Putonghua but widespread usage in Hong Kong, the same passage could be read out in both Cantonese and Putonghua without any modifications. The native Cantonese-speaking speakers providing the recordings were instructed to read out the passage verbatim and to avoid injecting any colloquial Cantonese words (e.g., replacing “是” with “係”; “is” in English) when reading out loud.

4 Perceived Outgroup Threat did not differ across Conditions, *t* = 0.76, *p* = 0.45, *95% CI* [−0.05, 0.11], Cohen’s *d* = 0.06.

5 The two threat subscales yielded a highly similar pattern of results when analysed separately (see Appendix G). For simplicity, the two subscale scores were collapsed to form a score of overall perceived threat.

6 Accent strength and ease of comprehension varied as expected across accent levels (see Appendix B). Analyses of accent levels separately yielded consistent findings for competence and comprehensibility ratings, but weakened effects for warmth and accent strength ratings (see Appendix H).

7 We also tested 4-way interaction models with speaker gender as an additional predictor. None of the 2-way, 3-way, and 4-way interactions that included both outgroup threat and speaker gender reached significance for any of the outcome variables, thus we proceeded with the main 3-way interaction model.

8 The inclusion of random slopes for Speaker led to model non-convergence for all variables, thus only random intercepts for Speaker were included in the final models.

9 A saturated path analysis model fit using the lavaan package in R yielded consistent results. We used the same collapsed dataset instead of multilevel data as lavaan currently does not support multilevel SEM with random slopes.

10 While we primarily focused on the serial mediation path, the moderating effect of Language Variety was tested for all three indirect paths (i.e., Accent Strength → Comprehensibility, Accent Strength, and Comprehensibility).

11 While all speakers were categorized with above-chance accuracy during pilot testing (see Appendix A), we cannot be certain whether the current participants correctly inferred each speaker’s social group.
