# Supplementary material for: Comprehension under threat: outgroup threat predicts perceived accent strength, ease of comprehension, and speaker evaluations
Source: Front Psychol. 2026 Oct 16;17:1835739. doi: 10.3389/fpsyg.2026.1835739 (PMC13719614; doi:10.3389/fpsyg.2026.1835739)
Supplement: Supplementary file 1 [file Supplementary_file_1.DOCX]

**Appendix A: Track Preparation**

The recording tracks used in the current study were created in two batches for two separate studies in 2013 and 2015 respectively. In each batch, a short passage (see below) was read out by each speaker in both Cantonese and Putonghua, creating recording tracks between 20 to 30 seconds in length.

*“為進一步推廣社區「普及體育」的文化，體育委員會自 2007 年起每兩年舉辦一屆運動會。運動會是一個以18區區議會為參賽單位的大型綜合運動會，舉辦目的是在社區層面提供更多體育參與、交流和合作的機會，鼓勵市民積極參與體育運動，從而提升地區體育水平。[In English: To further promote the "Sport for All" culture in the community, the Sports Commission has organised the Sports Games biennially since 2007. The Sports Games is a territory-wide major multi-sport event with the 18 District Councils (DCs) as participating units. The objectives of the Sports Games are to provide districts with more opportunities for sports participation, exchanges and co-operation, and to encourage active public participation in sports.]”*

The first batch of recordings were made by 4 female undergraduate students aged between 19-21 years old. Two students were native Cantonese speakers born and raised in Hong Kong. Two students were native Putonghua speakers from Mainland China who had studied in Hong Kong for 3-4 years. Speakers recorded the passage in a quiet environment and were asked to read out the passage in Cantonese and Putonghua multiple times. Two of the best tracks per speaker (one Cantonese and one Putonghua) were selected for pilot testing. Thirty-three undergraduate judges rated the eight tracks on accent “standardness” (1 = Not at all standard; 7 = Extremely standard) and guessed, out of three choices (“Hongkonger”, “Mainlander”, “Other Ethnicities”), the ethnicity of each speaker.

The second batch of recordings were made by 11 speakers (5 Hong Kong-Chinese, 6 Mainland-Chinese; aged 18-21) in both Cantonese and Putonghua. Speakers recorded the passage in a sound-attenuated room and were asked to read out the passage multiple times. Two tracks per speaker (one Cantonese and one Putonghua) were selected for pilot testing. Two pilot studies were conducted (N=27 and N=28 respectively). Undergraduate judges rated each speaker on accent strength (0 = No accent at all; 5 = Extremely strong accent) and guessed, out of three choices (“Hong Kong”, “Mainland China”, “Other locations”) where each speaker came from. All speaker characteristics are presented in table S1.

**Table S1**

*Pilot Test Accent Strength Ratings and Identification Rates of the Final Set of Putonghua and Cantonese Speakers*

| Putonghua | | | | | |
| --- | --- | --- | --- | --- | --- |
| Speaker | Condition | Gender | Ethnicity | Accent Strength | % Correctly Identified |
| D1 | Native | Male | Mainland-Chinese | 0.32 | 82.14% |
| C1 | Mild Non-native | Male | Hong Kong-Chinese | 2.30 | 88.89% |
| H1 | Moderate Non-native | Male | Hong Kong-Chinese | 3.19 | 92.86% |
| A1 | Strong Non-native | Male | Hong Kong-Chinese | 3.41 | 92.31% |
| E1 | Native | Female | Mainland-Chinese | 0.07 | 92.86% |
| B1 | Mild Non-native | Female | Hong Kong-Chinese | 1.37 | 71.43% |
| F1 | Moderate Non-native | Female | Hong Kong-Chinese | 2.35^a^ | 83.87% |
| G1 | Strong Non-native | Female | Hong Kong-Chinese | 3.81^a^ | 94.44% |
| Cantonese | | | | | |
| Speaker | Condition | Gender | Ethnicity | Accent Strength | % Correctly Identified |
| C2 | Native | Male | Hong Kong-Chinese | 0.30 | 92.00% |
| D2 | Mild Non-native | Male | Mainland-Chinese | 2.07 | 75.00% |
| H2 | Moderate Non-native | Male | Mainland-Chinese | 3.04 | 92.86% |
| A2 | Strong Non-native | Male | Mainland-Chinese | 3.36 | 89.29% |
| B2 | Native | Female | Hong Kong-Chinese | 0.07 | 92.86% |
| G2 | Mild Non-native | Female | Mainland-Chinese | 2.22^a^ | 65.63% |
| E2 | Moderate Non-native | Female | Mainland-Chinese | 3.59 | 77.78% |
| F2 | Strong Non-native | Female | Mainland-Chinese | 3.93 | 96.43% |

Note. ^a^ Speakers F1, G1, and G2 were rated on accent “standardness” instead of accent strength. Their scores were reverse-scored and rescaled to create a comparable accent strength score.

**Appendix B: Speech Perception by Accent Level**

To compare accent strength and comprehensibility, we conducted mixed-model ANOVAs using the *rstatix* package (Kassambara, 2023) in *R 4.4.0* (R Core Team, 2024) with ratings (Level 1) nested within participants (Level 2) with Accent Level (Native, Mild Non-native, Moderate Non-native, Strong Non-native), Condition (Cantonese vs. Putonghua), and their interaction as the fixed effects. We specified random intercepts for participants.

The main effect of Condition was significant for both accent strength, *F*(1,5824) = 266.67, *p*<.0001, and ease of comprehension, *F*(1,5824) =516.95, *p*<.0001. The main effect of Accent Level was also significant for both accent strength, *F*(3,5824) = 2558.45, *p*<.0001, and ease of comprehension, *F*(3,5824) =2760.37, *p*<.0001. The two-way Condition x Accent Level interaction was significant for both accent strength, *F*(3,5824) =144.52, *p*<.0001, and ease of comprehension, *F*(1,5824) =165.17, *p*<.0001. Estimated marginal means and simple main effects are presented in Table S1.

***Table S2***

*Accent Strength and Comprehensibility Scores of Native-, Mild Non-native-, Moderate Non-native- and Strong Non-native-accented Speakers*

| **Accent Strength** | | | | | |
| --- | --- | --- | --- | --- | --- |
|  | **Native** | **Mild Non-native** | **Moderate Non-native** | **Strong Non-native** | ***F*** |
| **Cantonese** | 1.68 (.03)^a^ | 3.38 (.03)^b^ | 4.13 (.03)^c^ | 4.44 (.03)^d^ | 1919.13*** |
| **Putonghua** | 2.03 (.03)^a^ | 2.63 (.03)^b^ | 3.47 (.03)^c^ | 4.14 (.03)^d^ | 906.45*** |
| ***F*** | 59.18*** | 288.54*** | 282.703*** | 66.95*** |  |
| **Comprehensibility** | | | | | |
|  | **Native** | **Mild Non-native** | **Moderate Non-native** | **Strong Non-native** | ***F*** |
| **Cantonese** | 5.41 (.03)^a^ | 4.05 (.03)^b^ | 3.44 (.03)^c^ | 3.07 (.03)^d^ | 1608.76*** |
| **Putonghua** | 5.28 (.03)^a^ | 4.87 (.03)^b^ | 4.18 (.03)^c^ | 3.26 (.03)^d^ | 1308.68*** |
| ***F*** | 15.07*** | 481.18*** | 420.81*** | 30.91*** |  |

Note.	**** p<.001, ** p<.01, p<.05**

Different subscripts in each row indicate significant differences (Bonferroni adjusted)

**Appendix C: Full Moderated Mediation Analyses Results**

***Table S3***

*Summary of Regression Results for Models Predicting Ratings of Speaker Competence, Warmth, Accent Strength, and Ease of Comprehension (Non-Native Speakers only)*

|  | Accent Strength | Compre-hensibility | | Competence | | | Warmth |
| --- | --- | --- | --- | --- | --- | --- | --- |
| Fixed Effects | | | | | | | |
|  | *B (SE)* | *B (SE)* | | *B (SE)* | | | *B (SE)* |
| *(Intercept)* | 3.94***  (.34) | 4.97***  (.26) | | 1.54***  (.35) | | | 1.64***  (.42) |
| Language Variety (Putonghua) | -.57***  (.04) | .27***  (.03) | | -.15***  (.03) | | | -.15***  (.04) |
| Outgroup Threat | .17**  (.05) | -.20**  (.04) | | -.06  (.04) | | | .03  (.05) |
| Language x Threat | -.16*  (.07) | .18***  (.05) | | .02  (.05) | | | .02  (.06) |
| Accent Strength | -- | -.53***  (.03) | | -.13***  (.04) | | | -.05  (.04) |
| Ease of Comprehension | -- | -- | | .41***  (.03) | | | .33***  (.05) |
| *Control Variables* |  | |  | |  |  | |
| Age | -.01  (.01) | .02**  (.01) | | -.01  (.01) | | | -.02  (.01) |
| Gender (Man) | -.04  (.04) | .03  (.03) | | -.02  (.03) | | | -.07  (.04) |
| Dataset (2019) | -.03  (.05) | -.02  (.03) | | .04  (.04) | | | .01  (.04) |
| Dataset (2020) | -.02  (.05) | -.02  (.03) | | .07*  (.03) | | | .05  (.04) |
| Cantonese Proficiency | -.004  (.04) | -.05*  (.03) | | -.03  (.03) | | | .05  (.03) |
| Putonghua Proficiency | .02  (.03) | .08***  (.02) | | .06*  (.03) | | | .07*  (.03) |
| Cantonese Accentedness | -.10*  (.04) | -.05*  (.03) | | .08**  (.03) | | | .04  (.03) |
| Putonghua Accentedness | .06  (.04) | .02  (.03) | | .07*  (.03) | | | .09**  (.03) |
| Hongkonger Identity | .01  (.03) | .04  (.02) | | .02  (.02) | | | .02  (.03) |
| Chinese Identity | .05**  (.02) | .01  (.01) | | -.00  (.01) | | | .00  (.02) |

*Note. *** <.001, ** <.01, <.05**

**Appendix D: SEM Results**

A fully saturated path model was fit using *lavaan* (Rosseel, 2012) in R 4.4.0 (R Core Team, 2024). Since the model was just identified, the model fit the data perfectly, χ^2^(0) = 0, *p*=1.00. Bootstrapping with 5000 samples was used to estimate the standard errors and confidence intervals for all paths and indirect effects. The index of moderated mediation for the hypothesized serial mediation path (Threat -> Accent -> Comprehension -> Outcome) was significant for both Competence, *B*=.04, *SE* = .02, 95% CI [.004, .07], *z* = 2.15, *p*=.032, and Warmth, *B* = .03, *SE* = .01, 95% CI [.01, .06], *z* = 2.09, *p*=.037.

***Table S4***

*Summary of Regression Results for Models Predicting Ratings of Speaker Competence, Warmth, Accent Strength, and Comprehensibility (Non-Native Speakers only)*

|  | Accent Strength | Compre-hensibility | | Competence | | | Warmth |
| --- | --- | --- | --- | --- | --- | --- | --- |
| Fixed Effects | | | | | | | |
|  | *B (SE)* | *B (SE)* | | *B (SE)* | | | *B (SE)* |
| *(Intercept)* | 3.87***  (.39) | 5.03***  (.26) | | 1.51***  (.43) | | | 1.51**  (.49) |
| Language Variety (Putonghua) | -.57***  (.04) | .27***  (.03) | | -.15***  (.04) | | | -.16***  (.04) |
| Outgroup Threat | .17**  (.06) | -.20**  (.04) | | -.06  (.05) | | | .03  (.06) |
| Language x Threat | -.16*  (.07) | .18***  (.05) | | .02  (.06) | | | .02  (.07) |
| Accent Strength | -- | -.53***  (.03) | | -.13***  (.04) | | | -.05  (.05) |
| Ease of Comprehension | -- | -- | | .42***  (.05) | | | .33***  (.06) |
| *Control Variables* |  | |  | |  |  | |
| Age | -.01  (.01) | .03*  (.01) | | -.01  (.01) | | | -.02  (.01) |
| Gender (Man) | -.03  (.04) | .03  (.03) | | -.02  (.03) | | | -.07  (.04) |
| Dataset (2019) | -.03  (.05) | -.02  (.03) | | .04  (.03) | | | .01  (.04) |
| Dataset (2020) | -.02  (.04) | -.02  (.03) | | .07*  (.03) | | | .05  (.04) |
| Cantonese Proficiency | -.01  (.04) | -.06*  (.03) | | -.03  (.03) | | | .05  (.04) |
| Putonghua Proficiency | .02  (.04) | .08**  (.03) | | .06  (.03) | | | .07*  (.03) |
| Cantonese Accentedness | -.10*  (.04) | -.05  (.03) | | .08*  (.03) | | | .04  (.04) |
| Putonghua Accentedness | .06  (.04) | .02  (.03) | | .07*  (.03) | | | .09*  (.04) |
| Hongkonger Identity | .01  (.04) | .04*  (.02) | | .02  (.03) | | | .02  (.04) |
| Chinese Identity | .05**  (.02) | .01  (.01) | | -.00  (.02) | | | .00  (.02) |

*Note. *** <.001, ** <.01, <.05**

**Appendix E: Main Analyses Controlling for Covariates**

**Measures**

**Umbrella Movement Endorsement.** Participants responded to a single item “*“How much do you endorse the Umbrella/Occupy Central movement?”* on a 5-point Likert scale *(1 = “extremely against”; 5 = “extremely endorse”)*. This variable was measured in the 2018-2019 datasets only.

**Results**

The same multi-level regression models reported in the paper were re-run controlling only for participants’ demographics, dataset, and speaker gender (Model 1), participants’ language proficiencies (Model 2), social identification (Model 3), umbrella movement endorsement (Model 4), and all variables simultaneously (Model 5). The critical 3-way Threat × Speaker Accent × Condition interactions remain significant for Competence, Warmth, Accent Strength, and Ease of Comprehension (see Tables S5A – S5D) across all models.

***Table S5A***

*Summary of Multi-level Regression Results for Models Predicting Ratings of Speaker Competence, Controlling for Participants’ Language Proficiencies, Social Identification, and Umbrella Movement Endorsement*

|  | *(1)* | *(2)* | *(3)* | *(4)* | *(5)* |
| --- | --- | --- | --- | --- | --- |
|  | *B (SE)* | *B (SE)* | *B (SE)* | *B (SE)* | *B (SE)* |
| *(Intercept)* | 3.86 (.16)*** | 3.42 (0.23)*** | 3.76 (0.20)*** | 3.67 (0.21)*** | 3.21 (0.33)*** |
| Accent (Non-native) | -1.09 (.03)*** | -1.09 (0.03)*** | -1.09 (0.03)*** | -1.08 (0.05)*** | -1.08 (0.05)*** |
| Language Variety (Putonghua) | .15 (.04)*** | 0.14 (0.04)*** | 0.14 (0.04)*** | 0.15 (0.06)** | 0.15 (0.06)** |
| Outgroup Threat | .12 (.06)* | 0.14 (0.06)* | 0.11 (0.06)+ | 0.13 (0.08)+ | 0.15 (0.08)+ |
| Accent x Language | .01 (.05) | 0.01 (0.05) | 0.01 (0.05) | -0.01 (0.06) | -0.01 (0.06) |
| Language x Threat | -.16 (.08)* | -0.17 (0.08)* | -0.15 (0.08)* | -0.26 (0.10)* | -0.26 (0.10)** |
| Accent x Threat | -.32 (.06)*** | -0.32 (0.06)*** | -0.32 (0.06)*** | -0.42 (0.09)*** | -0.42 (0.09)*** |
| Accent x Language x Threat | .32 (.09)*** | 0.32 (0.09)*** | 0.32 (0.09)*** | 0.49 (0.12)*** | 0.49 (0.12)*** |
| *Control Variables* |  |  |  |  |  |
| Age | -.004 (.01) | -0.01 (0.01) | -0.00 (0.01) | -0.00 (0.01) | 0.00 (0.01) |
| Gender (Woman) | -.01 (.03) | -0.02 (0.03) | -0.02 (0.03) | 0.01 (0.04) | -0.00 (0.04) |
| Dataset (2019) | .05 (.04) | 0.06 (0.04)+ | 0.05 (0.04) | 0.03 (0.04) | 0.04 (0.04) |
| Dataset (2020) | .07 (.03)* | 0.08 (0.03)* | 0.08 (0.03)* |  |  |
| Speaker Gender (Male) | -.06 (.02)** | -0.06 (0.02)** | -0.06 (0.02)** | -0.06 (0.03)* | -0.06 (0.03)* |
|  |  |  |  |  |  |
| *Language Proficiencies* |  |  |  |  |  |
| Cantonese Proficiency |  | 0.01 (0.03) |  |  | -0.06 (0.04) |
| Putonghua Proficiency |  | 0.07 (0.02)** |  |  | 0.06 (0.03)* |
| Cantonese Accentedness |  | 0.08 (0.03)** |  |  | 0.08 (0.04)* |
| Putonghua Accentedness |  | 0.05 (0.03)+ |  |  | 0.02 (0.03) |
|  |  |  |  |  |  |
| *Social Identification* |  |  |  |  |  |
| Hong Kong Identity |  |  | 0.01 (0.01) |  | 0.04 (0.02)+ |
| Chinese Identity |  |  | 0.00 (0.01) |  | 0.01 (0.01) |
|  |  |  |  |  |  |
| Umbrella Movement Endorsement |  |  |  | 0.04 (0.02)* | 0.05 (0.02)* |
| Obs. | 5528 | 5528 | 5528 | 3368 | 3368 |

*Note. *** <.001, ** <.01, *<.05*

***Table S5B***

*Summary of Multi-level Regression Results for Models Predicting Ratings of Speaker Warmth, Controlling for Participants’ Language Proficiencies, Social Identification, and Umbrella Movement Endorsement*

|  | *(1)* | *(2)* | *(3)* | *(4)* | *(5)* |
| --- | --- | --- | --- | --- | --- |
|  | *B (SE)* | *B (SE)* | *B (SE)* | *B (SE)* | *B (SE)* |
| *(Intercept)* | 3.75 (.19)*** | 3.12 (0.26)*** | 3.61 (0.23)*** | 3.83 (0.24)*** | 3.38 (0.38)*** |
| Accent (Non-native) | -.43 (.03)*** | -0.43 (0.03)*** | -0.43 (0.03)*** | -0.44 (0.05)*** | -0.44 (0.05)*** |
| Language Variety (Putonghua) | -.27 (.05)*** | -0.28 (0.05)*** | -0.28 (0.05)*** | -0.27 (0.06)*** | -0.28 (0.06)*** |
| Outgroup Threat | .06 (.06) | 0.06 (0.06) | 0.07 (0.06) | 0.07 (0.08) | 0.07 (0.09) |
| Accent x Language | .34 (.05)*** | 0.34 (0.05)*** | 0.34 (0.05)*** | 0.32 (0.07)*** | 0.32 (0.07)*** |
| Language x Threat | -.20 (.09)* | -0.20 (0.09)* | -0.20 (0.09)* | -0.18 (0.11)+ | -0.18 (0.11)+ |
| Accent x Threat | -.11 (.06) | -0.11 (0.06)+ | -0.11 (0.06)+ | -0.18 (0.09)* | -0.18 (0.09)* |
| Accent x Language x Threat | .31 (.09)*** | 0.31 (0.09)*** | 0.31 (0.09)*** | 0.34 (0.12)** | 0.34 (0.12)** |
| *Control Variables* |  |  |  |  |  |
| Age | -.01 (.01) | -0.01 (0.01) | -0.01 (0.01) | -0.02 (0.01) | -0.02 (0.01) |
| Gender (Woman) | .03 (.03) | 0.02 (0.03) | 0.03 (0.03) | -0.02 (0.04) | -0.03 (0.04) |
| Dataset (2019) | .01 (.04) | 0.02 (0.04) | 0.01 (0.04) | 0.01 (0.04) | 0.02 (0.04) |
| Dataset (2020) | .02 (.04) | 0.04 (0.04) | 0.03 (0.04) |  |  |
| Speaker Gender (Male) | -.07 (.02)*** | -0.07 (0.02)*** | -0.07 (0.02)*** | -0.07 (0.02)** | -0.07 (0.02)** |
|  |  |  |  |  |  |
| *Language Proficiencies* |  |  |  |  |  |
| Cantonese Proficiency |  | 0.05 (0.03) |  |  | 0.00 (0.04) |
| Putonghua Proficiency |  | 0.06 (0.03)* |  |  | 0.05 (0.04) |
| Cantonese Accentedness |  | 0.06 (0.03)+ |  |  | 0.05 (0.04) |
| Putonghua Accentedness |  | 0.06 (0.03)+ |  |  | 0.04 (0.04) |
|  |  |  |  |  |  |
| *Social Identification* |  |  |  |  |  |
| Hong Kong Identity |  |  | 0.01 (0.02) |  | 0.02 (0.02) |
| Chinese Identity |  |  | 0.01 (0.01) |  | 0.01 (0.02) |
|  |  |  |  |  |  |
| Umbrella Movement Endorsement |  |  |  | 0.04 (0.02) | 0.04 (0.03) |
| Obs. | *5528* | 5528 | 5528 | 3368 | 3368 |

*Note. *** <.001, ** <.01, *<.05*

***Table S5C***

*Summary of Multi-level Regression Results for Models Predicting Ratings of Speaker Accent Strength, Controlling for Participants’ Language Proficiencies, Social Identification, and Umbrella Movement Endorsement*

|  | *(1)* | *(2)* | *(3)* | *(4)* | *(5)* |
| --- | --- | --- | --- | --- | --- |
|  | *B (SE)* | *B (SE)* | *B (SE)* | *B (SE)* | *B (SE)* |
| *(Intercept)* | 1.58 (.19)*** | 1.65 (0.27)*** | 1.55 (0.24)*** | 1.70 (0.26)*** | 1.73 (0.40)*** |
| Accent (Non-native) | 2.29 (.04)*** | 2.29 (0.04)*** | 2.29 (0.04)*** | 2.26 (0.06)*** | 2.26 (0.06)*** |
| Language Variety (Putonghua) | 0.36 (.05)*** | 0.36 (0.05)*** | 0.36 (0.06)*** | 0.38 (0.07)*** | 0.39 (0.07)*** |
| Outgroup Threat | -0.07 (.07) | -0.08 (0.07) | -0.05 (0.08) | -0.05 (0.10) | 0.01 (0.10) |
| Accent x Language | -0.93 (.06)*** | -0.93 (0.06)*** | -0.93 (0.06)*** | -0.92 (0.08)*** | -0.92 (0.08)*** |
| Language x Threat | 0.05 (.10) | 0.05 (0.10) | 0.05 (0.10) | 0.07 (0.13) | 0.06 (0.13) |
| Accent x Threat | 0.23 (.08)** | 0.23 (0.08)** | 0.23 (0.08)** | 0.31 (0.11)** | 0.31 (0.11)** |
| Accent x Language x Threat | -0.24 (.11)* | -0.24 (0.11)* | -0.24 (0.11)* | -0.38 (0.15)* | -0.38 (0.15)* |
| *Control Variables* |  |  |  |  |  |
| Age | .002 (.01) | 0.00 (0.01) | 0.00 (0.01) | 0.00 (0.01) | -0.00 (0.01) |
| Gender (Woman) | .01 (.03) | 0.02 (0.04) | 0.01 (0.04) | -0.00 (0.04) | 0.00 (0.04) |
| Dataset (2019) | -.06 (.04) | -0.07 (0.04) | -0.06 (0.04) | -0.06 (0.04) | -0.04 (0.04) |
| Dataset (2020) | -.09 (.04)* | -0.10 (0.04)* | -0.07 (0.04)+ |  |  |
| Speaker Gender (Male) | .22 (.02)*** | 0.22 (0.02)*** | 0.22 (0.02)*** | 0.25 (0.03)*** | 0.25 (0.03)*** |
|  |  |  |  |  |  |
| *Language Proficiencies* |  |  |  |  |  |
| Cantonese Proficiency |  | -0.03 (0.03) |  |  | 0.00 (0.05) |
| Putonghua Proficiency |  | -0.01 (0.03) |  |  | -0.00 (0.04) |
| Cantonese Accentedness |  | 0.00 (0.03) |  |  | 0.04 (0.04) |
| Putonghua Accentedness |  | 0.05 (0.03) |  |  | 0.07 (0.04) |
|  |  |  |  |  |  |
| *Social Identification* |  |  |  |  |  |
| Hong Kong Identity |  |  | -0.00 (0.02) |  | -0.04 (0.02)+ |
| Chinese Identity |  |  | 0.01 (0.01) |  | 0.02 (0.02) |
|  |  |  |  |  |  |
| Umbrella Movement Endorsement |  |  |  | -0.03 (0.03) | -0.03 (0.03) |
| Obs. | *5528* | 5528 | 5528 | 3368 | 3368 |

*Note. *** <.001, ** <.01, *<.05*

***Table S5D***

*Summary of Multi-level Regression Results for Models Predicting Ease of Comprehension, Controlling for Participants’ Language Proficiencies, Social Identification, and Umbrella Movement Endorsement*

|  | *(1)* | *(2)* | *(3)* | *(4)* | *(5)* |
| --- | --- | --- | --- | --- | --- |
|  | *B (SE)* | *B (SE)* | *B (SE)* | *B (SE)* | *B (SE)* |
| *(Intercept)* | 5.26 (0.16)*** | 5.00 (0.23)*** | 4.95 (0.20)*** | 5.15 (0.22)*** | 4.56 (0.33)*** |
| Accent (Non-native) | -1.89 (0.04)*** | -1.89 (0.04)*** | -1.89 (0.04)*** | -1.82 (0.05)*** | -1.82 (0.05)*** |
| Language Variety (Putonghua) | -0.14 (0.04)** | -0.14 (0.04)** | -0.14 (0.04)** | -0.13 (0.06)* | -0.14 (0.06)* |
| Outgroup Threat | 0.06 (0.06) | 0.07 (0.06) | 0.05 (0.06) | 0.09 (0.08) | 0.10 (0.08) |
| Accent x Language | 0.71 (0.05)*** | 0.71 (0.05)*** | 0.71 (0.05)*** | 0.65 (0.06)*** | 0.65 (0.07)*** |
| Language x Threat | -0.11 (0.08) | -0.10 (0.08) | -0.10 (0.08) | -0.14 (0.10) | -0.13 (0.10) |
| Accent x Threat | -0.34 (0.07)*** | -0.34 (0.07)*** | -0.34 (0.07)*** | -0.38 (0.09)*** | -0.38 (0.09)*** |
| Accent x Language x Threat | 0.38 (0.09)*** | 0.38 (0.09)*** | 0.38 (0.09)*** | 0.44 (0.12)*** | 0.44 (0.12)*** |
| *Control Variables* |  |  |  |  |  |
| Age | 0.01 (0.01) | 0.01 (0.01) | 0.01 (0.01)+ | 0.01 (0.01) | 0.02 (0.01)+ |
| Gender (Woman) | -0.03 (0.03) | -0.04 (0.03) | -0.03 (0.03) | -0.02 (0.04) | -0.04 (0.04) |
| Dataset (2019) | 0.03 (0.04) | 0.03 (0.04) | 0.04 (0.04) | 0.01 (0.04) | 0.02 (0.04) |
| Dataset (2020) | 0.05 (0.03) | 0.07 (0.03)* | 0.06 (0.03)+ |  |  |
| Speaker Gender (Male) | -0.18 (0.02)*** | -0.18 (0.02)*** | -0.18 (0.02)*** | -0.20 (0.03)*** | -0.20 (0.03)*** |
|  |  |  |  |  |  |
| *Language Proficiencies* |  |  |  |  |  |
| Cantonese Proficiency |  | 0.03 (0.03) |  |  | -0.01 (0.04) |
| Putonghua Proficiency |  | 0.06 (0.02)* |  |  | 0.06 (0.03)+ |
| Cantonese Accentedness |  | -0.01 (0.03) |  |  | -0.03 (0.04) |
| Putonghua Accentedness |  | -0.01 (0.03) |  |  | -0.03 (0.03) |
|  |  |  |  |  |  |
| *Social Identification* |  |  |  |  |  |
| Hong Kong Identity |  |  | 0.03 (0.01)* |  | 0.04 (0.02)+ |
| Chinese Identity |  |  | 0.02 (0.01)+ |  | 0.03 (0.01)* |
|  |  |  |  |  |  |
| Umbrella Movement Endorsement |  |  |  | 0.03 (0.02) | 0.06 (0.02)* |
| Obs. | *5528* | 5528 | 5528 | 3368 | 3368 |

*Note. *** <.001, ** <.01, *<.05*

The same moderated-mediation model was tested using the PROCESS macro (model 85; Hayes, 2022) in R (version 4.4.0) while controlling for language proficiencies and social identification (model 1; full sample) and all three sets of covariates (model 2; 2018-2019 datasets only). The critical index of moderated mediation for the hypothesized serial mediation path (Threat 🡪 Accent 🡪 Comprehension 🡪 Outcome) remains significant for both Competence, and Warmth across both models (see Tables S6A - S6B)

**Table S6A**

*Index of Moderated Mediation and Conditional Indirect Effects by Language Variety, Controlling for Language Proficiencies and Social Identification*

| Mediator(s) | Outcome | *Index of Mediated Moderation (SE)* | *95% CI* | Conditional Indirect Effects | | | | |
| --- | --- | --- | --- | --- | --- | --- | --- | --- |
|  |  |  |  | Cantonese | |  | Putonghua | |
|  |  |  |  | *B (SE)* | *95% CI* |  | *B (SE)* | *95% CI* |
| Accent -> Comprehension | Competence | **.03 (.02)** | **[.01, .07]** | **-.04 (.01)** | **[-.06, -.01]** |  | .001 (.01) | [-.02, .02] |
|  | Warmth | **.03 (.01)** | **[.004, .06]** | **-.03 (.01)** | **[-.05, -.01]** |  | .001 (.01) | [-.02, .02] |
| Accent | Competence | .02 (.01) | **[.002, .05]** | **-.02 (.01)** | **[-.04, -.004]** |  | -.001 (.01) | [-.02, .01] |
|  | Warmth | .01 (.01) | [-.01, .03] | -.01 (.01) | [-.03, .01] |  | -.000 (.004) | [-.01, .01] |
| Comprehension | Competence | **.07 (.02)** | **[.03, .12]** | **-.08 (.02)** | **[-.13, -.05]** |  | .01 (.02) | [-.04, .02] |
|  | Warmth | .06 (.02) | **[.02, .10]** | **-.06 (.02)** | **[-.10, -.03]** |  | -.01 (.01) | [-.03, .02] |

**Table S6B**

*Index of Moderated Mediation and Conditional Indirect Effects by Language Variety, Controlling for Language Proficiencies, Social Identification, and Umbrella Movement Endorsement*

| Mediator(s) | Outcome | *Index of Mediated Moderation (SE)* | *95% CI* | Conditional Indirect Effects | | | | |
| --- | --- | --- | --- | --- | --- | --- | --- | --- |
|  |  |  |  | Cantonese | |  | Putonghua | |
|  |  |  |  | *B (SE)* | *95% CI* |  | *B (SE)* | *95% CI* |
| Accent -> Comprehension | Competence | **.06 (.02)** | **[.02, .11]** | **-.06 (.02)** | **[-.11, -.03]** |  | -.003 (.01) | [-.03, .02] |
|  | Warmth | **.05 (.02)** | **[.01, .09]** | **-.05 (.02)** | **[-.09, -.02]** |  | -.002 (.01) | [-.03, .02] |
| Accent | Competence | **.03 (.02)** | **[.001, .07]** | **-.03 (.02)** | **[-.07, -.002]** |  | -.001 (.01) | [-.02, .01] |
|  | Warmth | .01 (.02) | [-.02, .05] | -.01 (.02) | [-.05, .02] |  | -.001 (.005) | [-.01, .01] |
| Comprehension | Competence | **.06 (.03)** | **[.01, .12]** | **-.05 (.03)** | **[-.11, -.01]** |  | .01 (.02) | [-.03, .04] |
|  | Warmth | .05 (.02) | **[.01, .10]** | **-.04 (.02)** | **[-.09, -.01]** |  | .004  (.02) | [-.03, .03] |

**Appendix F: Main Analyses with Dataset as Moderator**

Perceived outgroup threat differed across datasets, with significantly higher scores for the 2019 dataset, *B*=.13, *SE*=.05, *95% CI* [.03, .23], *t*=2.48, *p*=.01, and 2020 dataset, *B*=.13, *SE*=.05, *95% CI* [.03, .22], *t*=2.61, *p*=.01, than the 2018 dataset. However, perceived threat did not differ between 2019 and 2020 datasets, *B*=.005, *SE*=.05, *95% CI* [-.10, .09], *t*=-0.09, *p*=.93. The elevated threat scores in 2019 and 2020 is unsurprising, given the Anti-ELAB protests during that period.

To examine whether cross-dataset differences in perceived outgroup threat impacted the key threat x language variety x speaker accent interaction, we conducted multilevel linear regressions with ratings (Level 1) nested within participants (Level 2) and speaker (Level 2) and tested a four-way threat x language variety x speaker accent x Dataset interaction. Dataset was a categorical variable, with the 2018 dataset serving as reference. Full results are presented in Table S7.

The four-way interactions were not significant for warmth (2019: p=.32; 2020: p=.57), nor comprehensibility (2019: p=.21; 2020: p=.12), indicating that effects of the three-way interaction were somewhat homogenous for warmth and comprehensibility. However, while the key simple slopes trended in the same direction across all datasets for both variables, not all reached significance (see Table S8).

The four-way interactions were significant for competence (2019: p =.06; 2020: p = .002) and accent strength (2019: p =.01; 2020: p =.009), indicating the effect of the three-way interaction differed across datasets. Inspection of simple slopes showed that the effects of threat on competence and accent strength ratings of Putonghua-accented Cantonese speakers were significant only in the 2018 dataset (see Table S8).

***Table S7***

*Summary of Multilevel Regression Results for Supplementary Model (with Dataset as Moderator) Predicting Ratings of Speaker Competence, Warmth, Accent Strength and Comprehensibility*

|  | Competence | Warmth | Accent Strength | Compre-hensibility |
| --- | --- | --- | --- | --- |
| (Intercept) | 3.95 (0.17)*** | 3.73 (0.19)*** | 1.46 (0.20)*** | 5.23 (0.51)*** |
| Accent (Non-native) | -1.10 (0.21)*** | -0.38 (0.11)*** | 2.28 (0.27)*** | -1.82 (0.50)*** |
| Language Variety (Putonghua) | 0.09  (0.07) | -0.19 (0.08)* | 0.46 (0.10)*** | -0.17  (0.07)* |
| Outgroup Threat | 0.16  (0.11) | 0.09  (0.12) | -0.16  (0.14) | 0.10  (0.10) |
| Age | -0.00  (0.01) | -0.01  (0.01) | 0.00  (0.01) | 0.01  (0.01) |
| Gender (Woman) | -0.03  (0.03) | 0.03  (0.03) | 0.02  (0.04) | -0.04  (0.03) |
| Dataset (2019) | -0.00  (0.08) | 0.12  (0.09) | -0.03  (0.11) | 0.02  (0.08) |
| Dataset (2020) | 0.04  (0.07) | 0.06  (0.08) | -0.09  (0.09) | 0.13  (0.07)+ |
| Speaker Gender (Male) | -0.17 (0.03)*** | -0.17 (0.03)*** | 0.37 (0.03)*** | -0.18  (0.43) |
| Accent (Non-native) x Language Variety (Putonghua) | 0.02  (0.08) | 0.23 (0.08)** | -1.00 (0.11)*** | 0.71 (0.08)*** |
| Language Variety (Putonghua) x Outgroup Threat | -0.26  (0.14)+ | -0.26 (0.15)+ | 0.25  (0.18) | -0.11  (0.14) |
| Accent (Non-native) x Outgroup Threat | -0.60 (0.12)*** | -0.20  (0.12) | 0.48 (0.16)** | -0.49 (0.12)*** |
| Accent (Non-native) x Dataset (2019) | -0.01  (0.09) | -0.15  (0.09) | -0.00  (0.12) | -0.03  (0.09) |
| Accent (Non-native) x Dataset (2020) | -0.00  (0.08) | -0.04  (0.08) | 0.07  (0.10) | -0.16  (0.08)* |
| Language Variety (Putonghua) x Dataset (2019) | 0.12  (0.11) | -0.17  (0.12) | -0.16  (0.14) | 0.10  (0.11) |
| Language Variety (Putonghua) x Dataset (2020) | 0.04  (0.10) | -0.12  (0.11) | -0.14 (0.13) | 0.01  (0.10) |
| Outgroup Threat x Dataset (2019) | 0.01  (0.15) | -0.02  (0.16) | 0.16  (0.20) | 0.03  (0.15) |
| Outgroup Threat x Dataset (2020) | -0.10  (0.14) | -0.08  (0.15) | 0.09  (0.18) | -0.14  (0.13) |
| Accent (Non-native) x Language Variety (Putonghua) x Outgroup Threat | 0.71 (0.16)*** | 0.42 (0.16)** | -0.74 (0.20)*** | 0.58 (0.16)*** |
| Accent (Non-native) x Language Variety (Putonghua) x Dataset (2019) | -0.02  (0.12) | 0.23 (0.13)+ | 0.12  (0.16) | -0.09  (0.12) |
| Accent (Non-native) x Language Variety (Putonghua) x Dataset (2020) | 0.06  (0.11) | 0.16  (0.11) | 0.05  (0.14) | 0.08  (0.11) |
| Language Variety (Putonghua) x Outgroup Threat x Dataset (2019) | 0.00  (0.20) | 0.18  (0.22) | -0.37  (0.26) | -0.07  (0.20) |
| Language Variety (Putonghua) x Outgroup Threat x Dataset (2020) | 0.26  (0.19) | 0.02  (0.21) | -0.24  (0.25) | 0.04  (0.19) |
| Accent (Non-native) x Outgroup Threat x Dataset (2019) | 0.35  (0.17)* | 0.08  (0.17) | -0.33  (0.22) | 0.23  (0.17) |
| Accent (Non-native) x Outgroup Threat x Dataset (2020) | 0.42  (0.16)** | 0.18  (0.16) | -0.38 (0.20)+ | 0.24  (0.16) |
| Accent (Non-native) x Language Variety (Putonghua) x Outgroup Threat x Dataset (2019) | -0.44  (0.23)+ | -0.23  (0.23) | 0.74 (0.29)* | -0.28  (0.23) |
| Accent (Non-native) x Language Variety (Putonghua) x Outgroup Threat x Dataset (2020) | -0.69 (0.22)** | -0.13  (0.22) | 0.73 (0.28)** | -0.34  (0.22) |
| Obs. | 5528 | 5528 | 5528 | 5528 |
| R2 Marg. | 0.397 | 0.073 | 0.641 | 0.422 |
| AIC | 11360.5 | 11439.5 | 12373.3 | 10804.2 |
| BIC | 11592.1 | 11677.7 | 12598.3 | 11022.6 |

***Table S8***

*Simple slopes of perceived outgroup threat on ratings of Native Putonghua Speakers Putonghua-accented Cantonese speakers by Dataset*

|  | Native Putonghua speakers | | | | Putonghua-accented Cantonese speakers | | | | Native Cantonese Speakers | | | | Cantonese-accented Putonghua Speakers | | | |
| --- | --- | --- | --- | --- | --- | --- | --- | --- | --- | --- | --- | --- | --- | --- | --- | --- |
|  | Comp | Warm | Acc | CP | Comp | Warm | Acc | CP | Comp | Warm | Acc | CP | Comp | Warm | Acc | CP |
|  | *B(SE)* | *B(SE)* | *B(SE)* | *B(SE)* | *B(SE)* | *B(SE)* | *B(SE)* | *B(SE)* | *B(SE)* | *B(SE)* | *B(SE)* | *B(SE)* | *B(SE)* | *B(SE)* | *B(SE)* | *B(SE)* |
| 2018 | -0.10  (0.09) | -0.17^+^  (0.10) | 0.09  (0.12) | -0.003  (0.09) | -0.44^***^  (0.08) | -0.10  (0.09) | 0.32^**^  (0.09) | -0.39^***^  (0.08) | 0.16  (0.11) | 0.09  (0.12) | -0.16  (0.14) | 0.10  (0.10) | 0.02  (0.07) | 0.06  (0.08) | -0.16^*^  (0.08) | 0.09  (0.07) |
| 2019 | -0.09  (0.10) | -0.002  (0.11) | -0.12  (0.13) | -0.05  (0.10) | -0.08  (0.08) | -0.04  (0.09) | 0.15^+^  (0.09) | -0.13^+^  (0.08) | 0.17  (0.10) | 0.08  (0.11) | 0.01  (0.14) | 0.13  (0.10) | -0.06  (0.07) | 0.07  (0.08) | 0.03  (0.08) | -0.01  (0.07) |
| 2020 | 0.07  (0.10) | -0.23*  (0.11) | -0.05  (0.13) | -0.10  (0.09) | -0.11  (0.07) | -0.004  (0.07) | 0.03  (0.07) | -0.30^***^  (0.07) | 0.06  (0.09) | 0.01  (0.10) | -0.07 (0.11) | -0.04  (0.08) | -0.09  (0.07) | 0.05  (0.08) | 0.04  (0.08) | -0.11  (0.07) |

*Note. *** <.001, ** <.01, *<.05,* ^+^ *<.10; Comp = Competence; Warm = Warmth; Acc = Accent Strength; CP = Comprehensibility;*

**Appendix G: Separate Analyses of Realistic and Symbolic Threat**

To test whether realistic and symbolic threat had different effects on speaker ratings, we tested the multilevel regression model reported in the paper separately for realistic threat (RT) and symbolic threat (ST).

**Realistic Threat.** The three-way interaction (RT x Speaker Accent x Condition) was significant for competence, *B*=.26, *SE*=.07, *95% CI* [.12, .40], *t*(2147.26*)*=3.70, *p*<.001, warmth, *B*=.20, *SE*=.07, *95% CI* [.06, .34], *t*(687.00) = 2.79, *p*=.005, accent strength, *B*=-.23, *SE*=.09, *95% CI* [-.40, -.05], *t*(687.00) = -2.54, *p*=.01, and comprehensibility, *B*=.28, *SE*=.07, *95% CI* [.15, .42], *t*(687.00) =4.05, *p*<.001. Examination of simple contrasts show that realistic threat predicted lower competence, *B*=-.14, *SE*=.03, *95% CI* [-.21, -.08], *z* = -4.25, *p*<.001, higher accent strength, *B*=.12, *SE*=.04, *95% CI* [.05, .20], *z* = 3.20, *p*=.001, and lower comprehensibility ratings, *B*=-.18, *SE*=.03, *95% CI* [-.25 -.11], *z* = -5.26, *p*<.001, of Putonghua-accented Cantonese speakers. However, realistic threat did not significantly predict lower warmth of native Putonghua speakers, *B*=-09., *SE*=.05, *95% CI* [-.18, .003], *z* = -1.90, *p*=.058.

**Symbolic Threat.** The three-way interaction (ST x Speaker Accent x Condition) was significant for competence, *B*=.24, *SE*=.10, *95% CI* [.05, .43], *t*(2091.37*)*=2.49, *p*=.01, warmth, *B*=.35, *SE*=.10, *95% CI* [.16, .54], *t*(687.00) = 3.56, *p*<.001, and comprehensibility, *B*=.33, *SE*=.09, *95% CI* [.15, .42], *t*(687.00) =3.46, *p*<.001, but not for accent strength, *B*=-.10, *SE*=.12, *95% CI* [-.34, .14], *t*(687.00) = -0.85, *p*=.40. Examination of simple contrasts show that symbolic threat predicted lower competence, *B*=-.19, *SE*=.05, *95% CI* [-.28, -.10], *z* = -4.24, *p*<.001, higher accent strength, *B*=.13, *SE*=.05, *95% CI* [.03, .23], *z* = 2.49, *p*=.01, and lower comprehensibility ratings, *B*=-.32, *SE*=.05, *95% CI* [-.41 -.23], *z* = -6.99, *p*<.001, of Putonghua-accented Cantonese speakers. Furthermore, symbolic threat significantly predicted lower warmth of native Putonghua speakers, *B*=-.15, *SE*=.07, *95% CI* [-.25, -.03], *z* = -2.35, *p*=.02.

In summary, analyses of each type of threat separately yielded largely the same pattern of results as the main analysis where the two types of threat were combined.

**Table S9.**

*Summary of Multilevel Regression Results for Supplementary Model (with Realistic Threat) Predicting Ratings of Speaker Competence, Warmth, Accent Strength, and Ease of Comprehension*

|  | Competence | Warmth | Accent Strength | Compre-hensibility |
| --- | --- | --- | --- | --- |
| (Intercept) | 3.87 (0.16)*** | 3.78 (0.19)*** | 1.57 (0.52)** | 5.29 (0.51)*** |
| Accent (Non-native) | -1.09 (0.03)*** | -0.43 (0.09)*** | 2.29 (0.50)*** | -1.89 (0.50)*** |
| Language Variety (Putonghua) | 0.15 (0.04)*** | -0.27 (0.05)*** | 0.36 (0.06)*** | -0.14 (0.04)*** |
| Realistic Threat | 0.10  (0.04)* | 0.03 (0.05) | -0.04 (0.06) | 0.04 (0.04) |
| Age | -0.00  (0.01) | -0.01 (0.01) | 0.00 (0.01) | 0.01 (0.01) |
| Gender (Woman) | -0.01  (0.03) | 0.03 (0.03) | 0.01 (0.03) | -0.02 (0.03) |
| Dataset (2019) | 0.04  (0.04) | 0.01 (0.04) | -0.06 (0.04) | 0.02 (0.04) |
| Dataset (2020) | 0.07  (0.03)* | 0.02 (0.04) | -0.08 (0.04)* | 0.04 (0.03) |
| Speaker Gender (Male) | -0.06 (0.02)** | -0.17 (0.03)*** | 0.22 (0.44) | -0.18 (0.43) |
| Accent (Non-native) x Language Variety (Putonghua) | 0.01  (0.05) | 0.34 (0.05)*** | -0.93 (0.06)*** | 0.71 (0.05)*** |
| Language Variety (Putonghua) x Realistic Threat | -0.14*  (0.06) | -0.12+ (0.07) | 0.05 (0.08) | -0.10+ (0.06) |
| Accent (Non-native) x Realistic Threat | -0.25***  (0.05) | -0.08 (0.05) | 0.16* (0.06) | -0.22***  (0.05) |
| Accent (Non-native) x Language Variety (Putonghua) x Realistic Threat | 0.26***  (0.07) | 0.20**  (0.07) | -0.23*  (0.09) | 0.28***  (0.07) |
| Obs. | 5528 | 5528 | 5528 | 5528 |
| R2 Marg. | 0.289 | 0.069 | 0.456 | 0.388 |
| AIC | 13116.9 | 11384.3 | 12329.3 | 10771.4 |
| BIC | 13236.0 | 11516.7 | 12455.0 | 10890.5 |

*Note. *** <.001, ** <.01, *<.05,* ^+^ *<.10*

**Table S10**

*Summary of Multilevel Regression Results for Supplementary Model (with Symbolic Threat) Predicting Ratings of Speaker Competence, Warmth, Accent Strength, and Ease of Comprehension*

|  | Competence | Warmth | Accent Strength | Compre-hensibility |
| --- | --- | --- | --- | --- |
| (Intercept) | 3.85*** (0.16) | 3.73*** (0.25) | 1.61** (0.52) | 5.27*** (0.51) |
| Accent (Non-native) | -1.09*** (0.03) | -0.43* (0.17) | 2.30*** (0.50) | -1.89*** (0.50) |
| Language Variety (Putonghua) | 0.15*** (0.04) | -0.27*** (0.05) | 0.36*** (0.05) | -0.13*** (0.04) |
| Symbolic Threat | 0.07  (0.06) | 0.09  (0.06) | -0.09  (0.08) | 0.07  (0.06) |
| Age | -0.00  (0.01) | -0.01 (0.01) | 0.00  (0.01) | 0.01  (0.01) |
| Gender (Woman) | -0.02  (0.03) | 0.04  (0.03) | 0.01  (0.04) | -0.03  (0.03) |
| Dataset (2019) | 0.05  (0.04) | 0.00  (0.04) | -0.06  (0.04) | 0.03  (0.04) |
| Dataset (2020) | 0.08*  (0.03) | 0.02  (0.04) | -0.09* (0.04) | 0.06+  (0.03) |
| Speaker Gender (Male) | -0.06** (0.02) | -0.07 (0.15) | 0.22  (0.44) | -0.18  (0.43) |
| Accent (Non-native) x Language Variety (Putonghua) | 0.02  (0.05) | 0.34*** (0.05) | -0.93*** (0.06) | 0.71*** (0.05) |
| Language Variety (Putonghua) x Symbolic Threat | -0.10  (0.08) | -0.24** (0.09) | 0.01  (0.11) | -0.05  (0.08) |
| Accent (Non-native) x Symbolic Threat | -0.27*** (0.07) | -0.11 (0.07) | 0.21* (0.09) | -0.38*** (0.07) |
| Accent (Non-native) x Language Variety (Putonghua) x Symbolic Threat | 0.24*  (0.10) | 0.35*** (0.10) | -0.10  (0.12) | 0.33*** (0.09) |
| Obs. | 5528 | 5528 | 5528 | 5528 |
| R2 Marg. | 0.288 | 0.037 | 0.455 | 0.390 |
| AIC | 13119.5 | 11373.2 | 12330.6 | 10747.7 |
| BIC | 13238.7 | 11492.3 | 12456.3 | 10866.8 |

*Note. *** <.001, ** <.01, *<.05,* ^+^ *<.10*

**Appendix H: Main Analyses by Accent Levels**

To test whether the effects found in the main analyses (where speakers with mild, moderate, and strong nonnative accents were collapsed into one category) were consistent across accent levels, we re-analysed the data with Speaker Accent as a four-level factor (native, mild nonnative, moderate nonnative, strong nonnative), with “native” as the reference category.

**Competence.** The critical three-way interaction was significant for mild, *B*=.34, *SE*=.08, *95% CI* [.17, .50], *t*(827.01) = 4.04, *p*<.001, moderate, *B*=.38, *SE*=.10, *95% CI* [.18, .58], *t*(761.23) = 3.73, *p*<.001, and strong, *B*=.25, *SE*=.12, *95% CI* [.006, .49], *t*(687.02) = 2.01, *p*=.04, nonnative-accented speakers. Analyses of simple slopes show that threat predicted lower competence ratings of Cantonese speakers with mild, *B*=-.15, *SE*=.05, *95% CI* [-.26, -.04], *z*=-2.73, *p*=.006, moderate, *B*=-.24, *SE*=.06, *95% CI* [-.35, -.13], *z*=-4.29, *p*<.001, and strong, *B*=-.21, *SE*=.06, *95% CI* [-.33, -.09], *z*=-3.47, *p*<.001, Putonghua accents. Threat also predicted higher competence ratings of native Cantonese speakers, *B*=.12, *SE*=.06, *95% CI* [.008, .23], *z*=2.10, *p*=.04.

**Warmth.** The critical three-way interaction was significant for mild, *B*=.27, *SE*=.09, *95% CI* [.10, .43], *t*(997.00) = 3.11, *p*=.002, and moderate, *B*=.47, *SE*=.10, *95% CI* [.27, .68], *t*(691.49) = 4.65, *p*<.001, nonnative-accented speakers, but not for strong nonnative-accented speakers, *B*=.19, *SE*=.12, *95% CI* [-.05, .43], *t*(690.24) = 1.56, *p*=.12. Analyses of simple slopes show that threat predicted lower warmth ratings of the native Putonghua speakers, *B*=-.14, *SE*=.06, *95% CI* [-.26, -.02], *z*=-2.30, *p*=.02.

**Accent Strength.** The critical three-way interaction was significant for mild nonnative-accented speakers, *B*=-.39, *SE*=.11, *95% CI* [-60, -.17], *t*(687.19) = -3.53, *p*<.001, but not for moderate, *B*=-.21, *SE*=.13, *95% CI* [-46, .04], *t*(716.48) = -1.65, *p*=.10, nor strong, *B*=-.12, *SE*=.14, *95% CI* [-40, .16], *t*(691.84) = -0.85, *p*=.40, nonnative-accented speakers. Analyses of simple slopes show that threat predicted higher accent strength ratings only for Cantonese speakers with mild, *B*=.16, *SE*=.07, *95% CI* [.03, .29], *z* = 2.38, *p*=.02, and moderate, *B*=.20, *SE*=.06, *95% CI* [.08, .32], *z* = 3.24, *p*=.001, Putonghua accents. Threat did not significantly predict accent strength ratings of Cantonese speakers with strong Putonghua accents, *B*=.11, *SE*=.06, *95% CI* [-.01, .23], *z* = 1.84, *p*=.07.

**Comprehensibility.** The critical three-way interaction was significant for mild, *B*=.43, *SE*=.09, *95% CI* [.26, .60], *t*(716.87) = 5.00, *p*<.001, moderate, *B*=.41, *SE*=.10, *95% CI* [.21, .61], *t*(746.63) = 4.02, *p*<.001, and strong, *B*=.30 *SE*=.12, *95% CI* [.07, .53], *t*(689.39) = 2.60, *p*=.01, nonnative-accented speakers. Analyses of simple slopes show that threat predicted lower comprehensibility ratings of Cantonese speakers with mild, *B*=-.26, *SE*=.06, *95% CI* [-.37, -.15], *z*=-4.57, *p*<.001, moderate, *B*=-.29, *SE*=.06, *95% CI* [-.40, -.18], *z*=-5.26, *p*<.001, and strong, *B*=-.30, *SE*=.05, *95% CI* [-.41, -.19], *z*=-5.43, *p*<.001, Putonghua accents.

In summary, the effects of threat on competence and comprehensibility ratings were consistent across all accent levels. However, the effects of threat on warmth and accent ratings were only partially consistent across accent levels. While direction of effects was consistent, the critical 3-way interaction failed to reach significance for strong nonnative-accented speakers (warmth and accent strength) and moderate nonnative-accented speakers (accent strength). Simple effects of threat on accent strength ratings of strong Putonghua-accented Cantonese speakers also failed to reach significance, indicating that the effects reported in the main analyses may lack robustness, especially for accent strength ratings.

**Table S11.**
*Summary of Multilevel Regression Results for Supplementary Model (with 4 Accent Levels) Predicting Ratings of Speaker Competence, Warmth, Accent Strength, and Comprehensibility*

|  | Competence | Warmth | Accent Strength | Compre-hensibility |
| --- | --- | --- | --- | --- |
| (Intercept) | 3.88*** (0.19) | 3.74*** (0.19) | 1.61*** (0.28) | 5.27*** (0.15) |
| Outgroup Threat | 0.12* (0.06) | 0.06 (0.06) | -0.07 (0.07) | 0.06 (0.05) |
| Language Variety (Putonghua) | 0.15*** (0.04) | -0.27*** (0.05) | 0.36*** (0.06) | -0.13** (0.04) |
| Age | -0.00 (0.01) | -0.01 (0.01) | 0.00 (0.01) | 0.01+ (0.01) |
| Gender (Woman) | -0.01 (0.03) | 0.03 (0.03) | 0.01 (0.03) | -0.02 (0.03) |
| Dataset (2019) | 0.04 (0.04) | 0.01 (0.04) | -0.06 (0.04) | 0.03 (0.03) |
| Dataset (2020) | 0.07* (0.03) | 0.03 (0.04) | -0.09* (0.04) | 0.06+ (0.03) |
| Speaker Gender (Male) | -0.06 (0.08) | -0.07*** (0.01) | 0.22 (0.19) | -0.29*** (0.02) |
| Accent (Mild) | -0.81*** (0.12) | -0.26*** (0.03) | 1.70*** (0.27) | -1.36*** (0.08) |
| Accent (Moderate) | -1.00*** (0.12) | -0.59*** (0.04) | 2.43*** (0.27) | -1.96*** (0.04) |
| Accent (Strong) | -1.44*** (0.13) | -0.44*** (0.05) | 2.76*** (0.28) | -2.34*** (0.29) |
| Outgroup Threat x Language Variety (Putonghua) | -0.16* (0.08) | -0.20* (0.09) | 0.05 (0.10) | -0.11 (0.08) |
| Outgroup Threat x Accent (Mild) | -0.27*** (0.06) | -0.10+ (0.06) | 0.23** (0.08) | -0.32*** (0.06) |
| Outgroup Threat x Accent (Moderate) | -0.36*** (0.07) | -0.19** (0.07) | 0.27** (0.09) | -0.35*** (0.07) |
| Outgroup Threat x Accent (Strong) | -0.33*** (0.09) | -0.04 (0.09) | 0.18+ (0.10) | -0.36*** (0.08) |
| Language Variety (Putonghua) x Accent (Mild) | 0.43*** (0.04) | 0.44*** (0.05) | -1.12*** (0.06) | 0.95*** (0.05) |
| Language Variety (Putonghua) x Accent (Moderate) | 0.05 (0.05) | 0.67*** (0.05) | -1.00*** (0.07) | 0.86*** (0.05) |
| Language Variety (Putonghua) x Accent (Strong) | -0.44*** (0.07) | -0.09 (0.07) | -0.67*** (0.08) | 0.32*** (0.06) |
| Outgroup Threat x Language Variety (Putonghua) x Accent (Mild) | 0.34*** (0.08) | 0.27** (0.09) | -0.39*** (0.11) | 0.43*** (0.09) |
| Outgroup Threat x Language Variety (Putonghua) x Accent (Moderate) | 0.38*** (0.10) | 0.47*** (0.10) | -0.21+ (0.13) | 0.41*** (0.10) |
| Outgroup Threat x Language Variety (Putonghua) x Accent (Strong) | 0.25* (0.12) | 0.19 (0.12) | -0.12 (0.14) | 0.30** (0.12) |
| Num.Obs. | 5528 | 5528 | 5528 | 5528 |
| R2 Marg. | 0.586 | 0.186 | 0.721 | 0.740 |
| AIC | 10653.3 | 10871.4 | 12014.4 | 10285.9 |
| BIC | 10871.7 | 11083.2 | 12232.8 | 10570.4 |

*Note. *** <.001, ** <.01, *<.05,* ^+^ *<.10*

**Appendix I: Full measures**

**Speaker Ratings**

1. According to your first impression, Speaker A/B… is:

(competent, confident, capable, skillful; friendly, warm, good-natured, sincere)

2. Please listen to speaker A/B… again and evaluate them.
1. I have to listen very closely to understand what the speaker says.
2. I find it hard to concentrate on what the speaker says because of the way s/he speaks.
3. The speaker speaks clearly.
4. I don't have any problems understanding the speaker.
5. The speaker’s Cantonese/Mandarin speaking ability is limited.
3. Speaker A/B has …:	no accent at all…… Has a very strong accent
4. Speaker A/B…’s accent is:	very standard……very non-standard

**Participant Measures**

***Language proficiency, accentedness, and language use***

1. The frequency with which you use **Cantonese** in your daily life is:	Never …… All the time
2. The frequency with which you use **Putonghua** in your daily life is:	Never …… All the time
3. Your **Cantonese** proficiency is:	Extremely low…… Extremely high
4. When you speak **Cantonese**, you have:	No accent at all……A very strong accent
5. Your **Cantonese** is:	Very Standard……Very non-standard
6. Your **Putonghua** proficiency is:	Extremely low…… Extremely high
7. When you speak **Putonghua**, you have:	No accent at all……A very strong accent
8. Your **Putonghua** is:	Very Standard……Very non-standard

***Perceived Outgroup Threat***

1. Mainland Chinese immigrants should learn to conform to the rules and norms of Hong Kong society as soon as possible after they arrive.
2. Immigration from Mainland China is undermining Hong Kong’s culture.
3. The values and beliefs of Mainland Chinese immigrants regarding work are basically quite similar to those of most Hongkongers.*
4. The values and beliefs of Mainland Chinese immigrants regarding moral and religious issues are not compatible with the beliefs and values of most Hongkongers.
5. The values and beliefs of Mainland Chinese immigrants regarding family issues and socializing children are basically quite similar to those of most Hongkongers.*
6. The values and beliefs of Mainland Chinese immigrants regarding social relations are not compatible with the beliefs and values of most Hongkongers.
7. Mainland Chinese immigrants should not have to accept Hong Kong’s ways.*
8. Mainland Chinese immigrants get more from this city than they contribute.
9. The children of Mainland Chinese immigrants should have the same right to attend public schools in Hong Kong as Hongkongers do.*
10. Mainland Chinese immigration has increased the tax burden on Hongkongers.
11. Mainland Chinese immigrants are not displacing Hong Kong workers from their jobs.*
12. Mainland Chinese immigrants should be eligible for the same health-care benefits received by Hongkongers.*
13. Social services have become less available to Hongkongers because of Mainland Chinese immigration.
14. The quality of social services available to Hongkongers has remained the same, despite Mainland Chinese immigration.*
15. Mainland Chinese immigrants are as entitled to subsidized housing or subsidized utilities (water, sewage, electricity) as poor Hongkongers are.*

***Social Identification***

You would describe yourself as a …

1. Hongkonger
2. Chinese person

(*1 = “extremely disagree; 5 = “extremely agree”*)
